# Mechanism of Iron-Catalyzed Oxidative α-Amination of Ketones with Sulfonamides

**DOI:** 10.1021/acs.joc.4c01401

**Published:** 2024-08-16

**Authors:** Gloria M. Parrales, Nina C. Hollin, Fubin Song, Yangyang Lyu, Anne-Marie O. Martin, Alexandra E. Strom

**Affiliations:** Department of Chemistry, Smith College, Northampton, Massachusetts 01063, United States

## Abstract

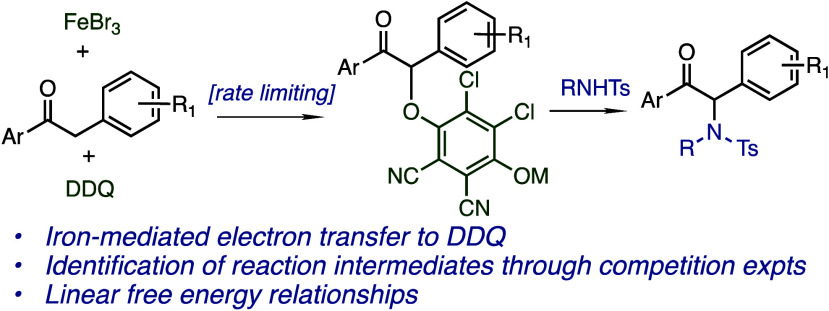

We report the mechanism of the iron-catalyzed oxidative α-amination of ketones with sulfonamides. Using linear free energy relationships, competition experiments, and identification of reaction intermediates, we have found that the mechanism of this reaction proceeds through rate-limiting electron transfer to 2,3-dichloro-5,6-dicyano-1,4-benzoquinone (DDQ) from an iron enolate in the process of forming an α-DDQ adduct. The adduct then serves as the electrophile for substitution with sulfonamide nucleophiles, accelerated by iron and additional DDQ. This mechanistic study rules out formation of an α-carbocation intermediate and purely radical mechanistic hypotheses.

## Introduction

Carbonyl compounds are ubiquitous and useful functional groups. The umpolung reactivity of the carbonyl functional group has captured the imagination of chemists, in particular α-umpolung reactivity.^1^ We recently reported the first oxidative α-amination of ketones with iron, which used 2,3-dichloro-5,6-dicyano-1,4-benzoquinone (DDQ) as the oxidant.^2^ This method was limited to sulfonamides as the nitrogen source and α-aryl (benzyl) ketones but demonstrated useful reactivity in the generation of aminated products from simple ketones and sulfonamides without prefunctionalization of either coupling partner. Herein, we propose a mechanism for this transformation that accounts for our observations.

Iron is earth-abundant, inexpensive, and readily available. The scientific community has specifically targeted iron catalysis as an area of intense focus and importance.^3,4^ Iron-catalyzed reactions of carbonyl compounds have resulted in a wide range of exciting products and mechanisms. Iron can function as a Lewis acid, catalyze cross-coupling reactions, and enable oxidation reactions, among other modes of catalysis.^5,6^

Our interest in the mechanism of our reported transformation stems, in part, from the role that DDQ plays in this reaction. Quinone-based oxidants can react through a variety of mechanisms,^7^ including inner sphere electron transfer and other covalent mechanisms, C–H abstraction, through photocatalytic activation,^8^ and through coordination directly to metal catalysts as a redox-active or ancillary ligand.^9^

**Scheme 1:**
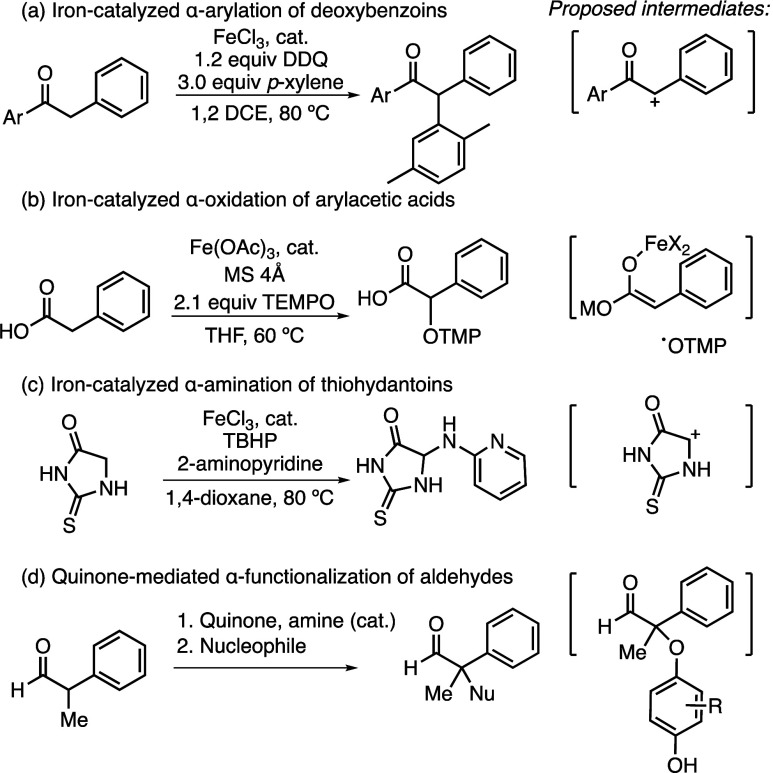
Mechanisms of α-Functionalization with Iron and DDQ

Oxidative iron catalysis has been shown to form α-functionalized products with a variety of carbonyl compounds and nucleophiles. The addition of TEMPO to arylacetic acids^10^ was found to proceed through the addition of the TEMPO radical to an iron(III) enolate, which can also be described as an iron(II) α-radical through resonance (Scheme 1a). Conversely, α-arylation of deoxybenzoins^11^ with iron(III) halide salts and DDQ is proposed to proceed through an α-carbocation (Scheme 1b), which has been observed in other systems, as well.^12^ A polar mechanism is proposed for this reaction, in part due to the distribution of products with aryl nucleophiles, which match Friedel–Crafts-type reactions. Oxidative iron catalysis has also been used to synthesize α-amino thiohydantoins^13^ through oxidation at the α-position to a stabilized carbocation (Scheme 1c). Our reported amination reaction could feature aspects of each of these mechanisms, given the high temperature and reaction conditions.

Given the reliance on quinone-based oxidants in our reported reaction, the quinone-mediated functionalization of carbonyl compounds is also relevant (Scheme 1d).^14^ This two-step reaction proceeds through an α-hydroquinone intermediate, which is then displaced by thiols and other nucleophiles. We hope that insight into the mechanism of the α-amination of ketones with DDQ, iron, and sulfonamides will allow us to expand the scope of coupling partners to other desirable umpolung functionalization reactions. Furthermore, insights into the formation and reactivity of the hydroquinone adduct intermediate have implications for organocatalysis and other modes of carbonyl activation with Lewis acids and oxidants.

## Results and Discussion

**Figure 1:**
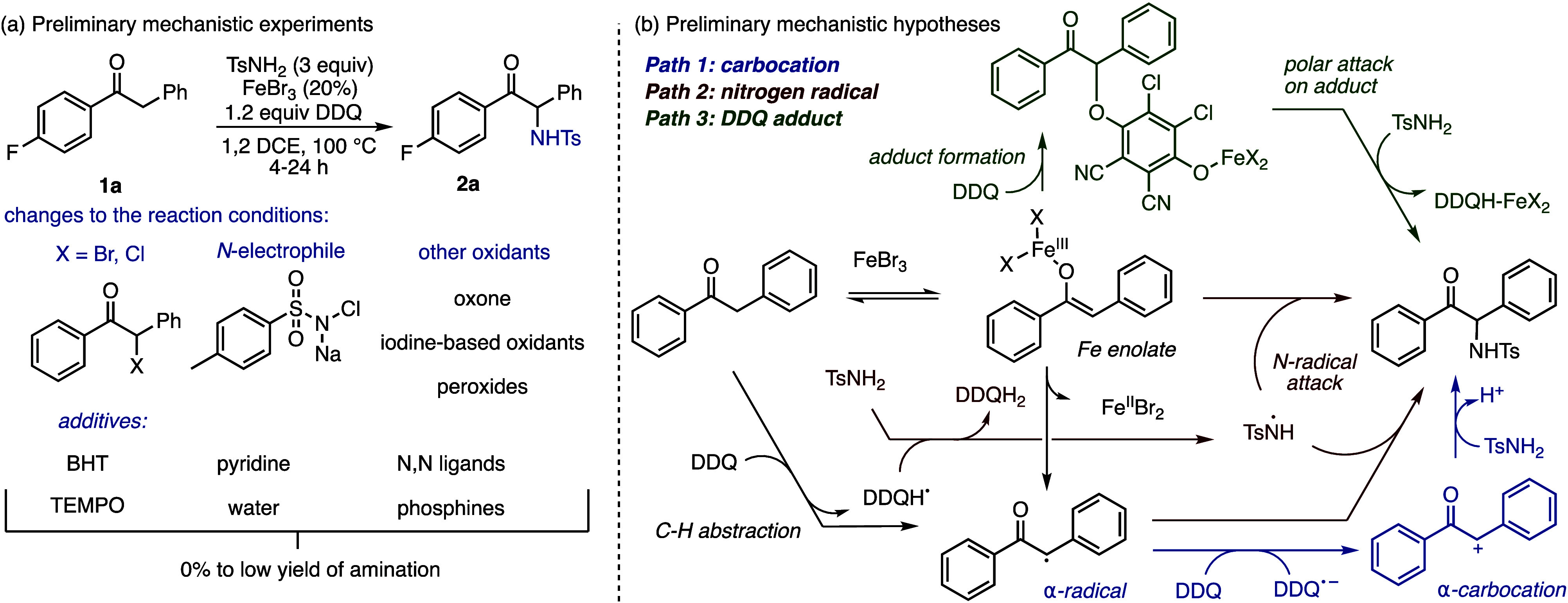
Preliminary mechanistic experiments and hypotheses.

Our exploration of the mechanism began with a series of trapping experiments. Addition of BHT and TEMPO to catalytic reaction mixtures led to only the recovered starting material (Figure 1a). Although this could point to a radical mechanism, we found that any coordinating additive (ligands, bases, etc.) stifled reactivity, as well, so we turned to reactions of isolated potential reaction intermediates for further insight. α-Halogenation or N-halogenation would result in electrophilic intermediates, and thus, we explored reactions of chloramine-T and α-chloro- and α-bromodeoxybenzoin. These reactions resulted in no or very low levels of product under a variety of reaction conditions. In addition, we explored alternate oxidants and found that while less oxidizing quinones gave low yields, non-quinone oxidants were ineffective for product formation. These initial experiments provided a few potential pathways for further consideration (Figure 1b). The reaction could proceed through stepwise C–H abstraction^15,16^ followed by oxidation of the ketone to generate an α-carbocation, which would be trapped with the sulfonamide to form the product (path 1), similar to mechanisms described in panels a and c of Scheme 1. This path is colored blue in Figure 1b. The formation of a sulfonamidyl radical could result in addition to an iron enolate or α-radical ketone intermediate (path 2), as shown in gray in Figure 1b. DDQ could also participate directly in the reaction through activation of the ketone by forming a transient quinol adduct (C- or O-bound),^17^ which could form a carbocation (heterolysis, with similarities to path 1), form an α-radical (homolysis, with similarities to path 2), or be displaced by a sulfonamide nucleophile (S_N_2) (path 3), as was proposed in the substitution reactions shown in Scheme 1d. This path is colored green in Figure 1b.

### Linear Free Energy Relationships

We chose to analyze the linear free energy relationship between substituents on the ketone and the formation of the product. While the independent collection of rate constants would be desirable, due to the presence of iron and high reaction temperatures, we used competition experiments to obtain relative rates of product formation.

**Figure 2:**
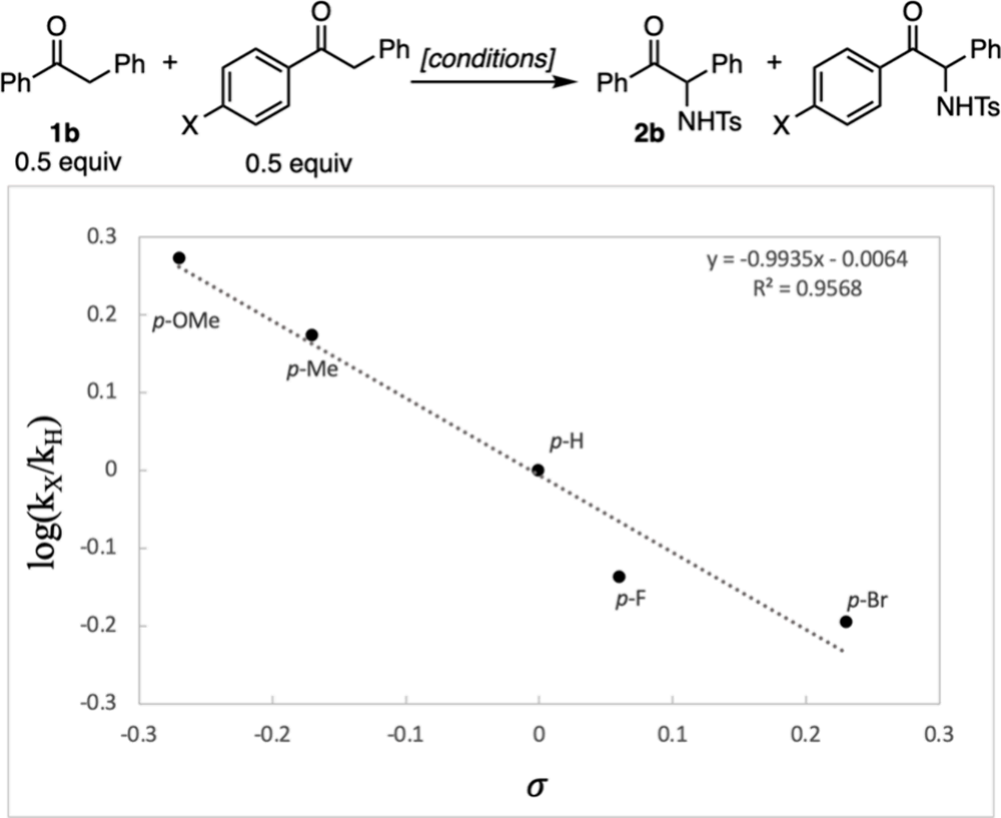
Hammett correlations for phenyl substitution.

**Figure 3:**
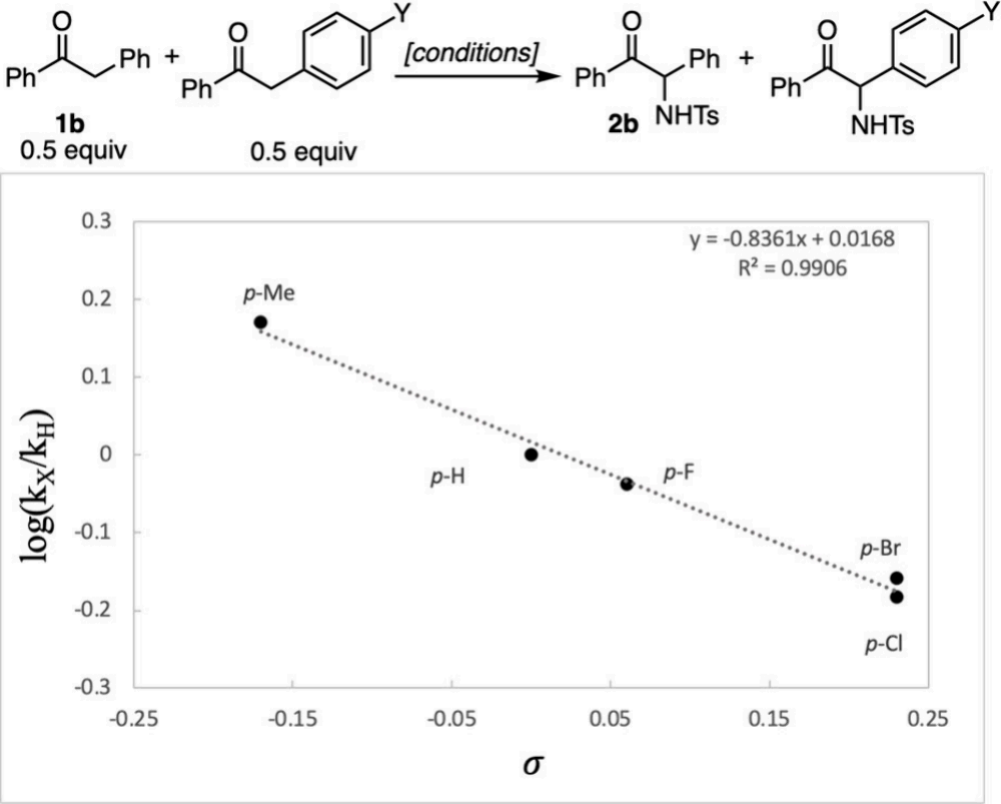
Hammett correlations for benzyl substitution.

The ρ values obtained for the phenyl [X (Figure 2)] and benzyl [Y (Figure 3)] substituents (−0.99 and −0.84, respectively) indicate a developing positive charge in the rate-determining step. Importantly, the phenyl and benzyl substituents both show that stabilization of positive charge enhances the rate with a slightly larger negative ρ value for the phenyl group. This suggests that the developing positive charge is spread out over both the carbonyl carbon and the α-carbon of an intermediate, such as an iron enolate or other π-system, with a larger developing positive charge at the carbonyl carbon. These linear free energy relationships allowed us to rule out radical formation as the rate-determining step, as this pathway would result in an increased rate for radical-stabilizing groups (see the Supporting Information for a comparison with radical parameters).

### Trapping of Potential Common Intermediates

The linear free energy relationships for both aryl groups inspired us to design experiments to access potential common intermediates through known reaction pathways, to compare the relative rates of product formation after the rate-determining step.^18,19^ The catalytic reaction was performed with deoxybenzoin **1b** as the ketone and a 1:1 mixture of excess *N*-methyl-*p*-toluenesulfonamide and *p*-toluenesulfonamide. A product ratio of 1.0:0.54 was found, favoring product formation from the *N*-alkyl sulfonamide (Table 1, entries 1 and 2). This ratio (consistent over a range of time points) serves as the basis for comparison with reactions of precursors to common intermediates (see the Supporting Information for competition experiments at different time points).

**Table 1:**
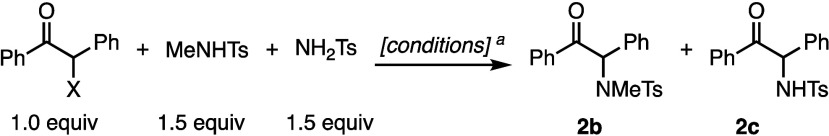
Competition Reactions

entry	substrate	reagent (equiv)	oxidant	time (min)	**2b**:**2c** ratio
1	**1b**	FeBr_3_ (0.2)	DDQ	6	1.00:0.54
2	**1b**	FeBr_3_ (0.2)	DDQ	10	1.00:0.57
3	**1c**	TMSOTf (0.2)	–	5	1.00:0.94
4	**1d**	FeBr_3_ (0.2)	DDQ	6	1.00:0.54
5^b^	**1e**	AgPF_6_ (1.2)	–	6	1.00:1.04

a Conditions: 0.2 M 1,2-dichloroethane, 100 °C. Time points were chosen to result in <15% conversion to **2b** and **2c**.

b Both products formed in high conversion (∼40%) in entry 5.

**Figure 4:**
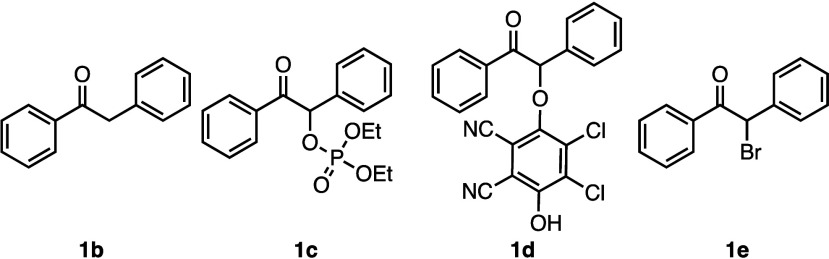
Precursors to reaction intermediates.

The α-carbocation precursors chosen for this study are α-phosphate ester **1c** and α-bromo derivatives of deoxybenzoin **1e** (Figure 4). These competition experiments were performed at the same temperature and in the same solvent as the catalytic reaction, to mimic the conditions as closely as possible. The α-ketophosphate is proposed to proceed through a carbocation (S_N_1) mechanism for substitution with a range of nucleophiles,^20^ and we found that TMSOTf was able to activate this electrophile for substitution with both *N*-methyl-*p*-toluenesulfonamide and *p*-toluenesulfonamide, each in 64% yield (see the Supporting Information for reactions with additional Lewis acids). The α-bromodeoxybenzoin could be activated with a silver(I) salt to form the same carbocation,^21^ although this activation was incredibly fast at the same temperature as our catalytic reaction (Table 1, entry 5). The ratios of reaction rates of secondary and primary sulfonamides are consistently ∼1:1 for these reactions, which allowed us to rule out product-determining steps that feature formation of a carbocation intermediate, either from oxidation of an α-radical intermediate or as an intermediate in reactions with a DDQ adduct (Table 1, entries 3 and 5).

We turned to an α-quinol adduct as an additional potential reaction intermediate [**1d** (Figure 4)]. These adducts have been synthesized from silyl enol ethers,^17^ formed as products from reactions of other carbonyl compounds,^22^ and proposed as intermediates in other α-functionalization reactions.^14^ The synthesis of this compound from the corresponding silyl enol ether followed by purification with column chromatography yielded the O-bound α-DDQ adduct. This adduct was then tested for product formation, which in the presence of catalytic iron(III) bromide gave low to moderate yields of the product. As our reaction mixture contains excess DDQ, we also tested for product formation in the presence of both iron and DDQ, which gave higher yields of the product. This increase in yield could be due to coordination to iron to further activate the DDQ adduct for displacement. Competition experiments with the DDQ adduct as the common intermediate precursor gave a 1.0:0.57 ratio (favoring the *N*-methyl sulfonamide), which, gratifyingly, matches the ratio of the competition in the catalytic reaction (Table 1, entry 4).

### Mechanistic Proposal

These results suggest that this amination reaction proceeds through formation of an O-bound α-DDQ adduct and not an α-radical or α-carbocation intermediate. The identified linear free energy relationship suggests that the rate-determining step of this reaction is the oxidation step en route to the α-DDQ adduct, because S_N_2 displacement would feature rate enhancement with electron-withdrawing groups. The magnitude of the linear free energy relationship is close to that observed for the generation of α-quinone adducts as determined by List et al.,^22^ which has been studied computationally.^24^ Importantly, although oxidation of the iron enolate forms a radical cation intermediate, the radical character at the α-position is likely conserved from the iron(III) enolate, and thus, oxidation leads to a transition state in which stabilization of the positive charge accelerates the reaction.

**Scheme 2:**
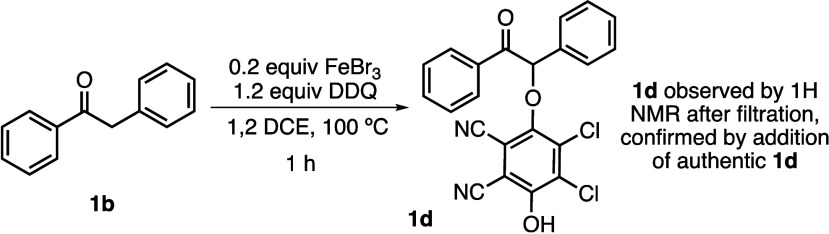
Formation of the Quinol Adduct with Iron and DDQ

We did not observe the proposed α-DDQ adduct in the course of the catalytic reaction. However, we hypothesized that by omitting the sulfonamide nucleophile, we may be able to isolate the intermediate formed in the reaction before it decomposed. Through modification of the reaction conditions, we were able to observe the conversion of ketone **1b** to adduct **1d**, the identity of which was confirmed by addition of independently prepared adduct **1d** (Scheme 2).

**Figure 5:**
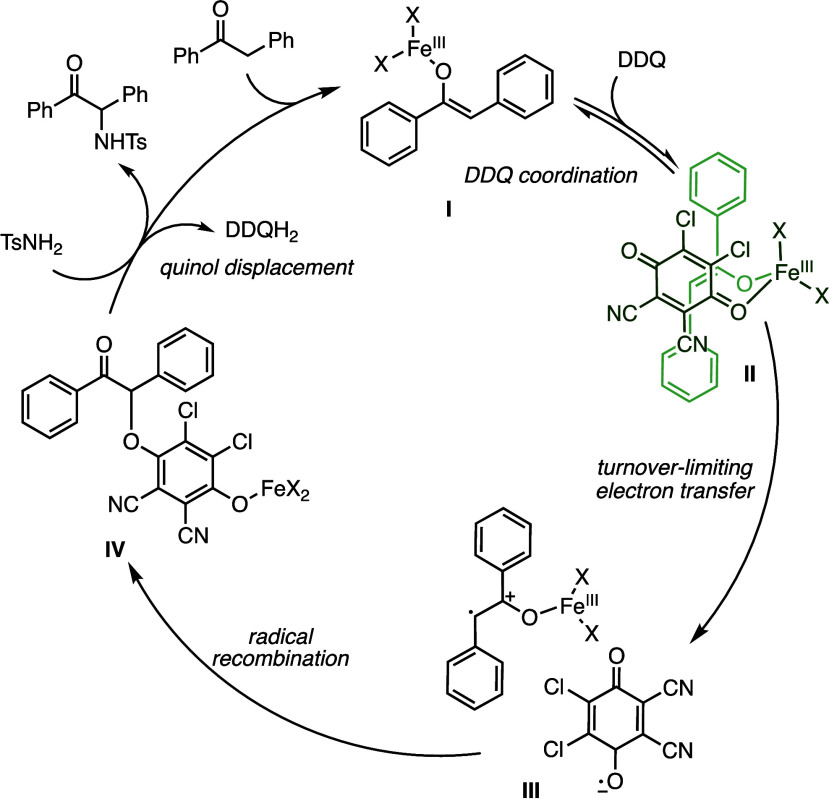
Proposed mechanism.

Thus, the proposed mechanism is shown in Figure 5. Rapid and reversible formation of iron enolate **I** is followed by reversible coordination of DDQ, facilitating turnover-limiting inner sphere electron transfer^23^ to form radical ion pairs **III**, which could maintain coordination through iron, or separate, as shown in Figure 5. These radical ions can recombine to form quinol adduct **IV**, which can be substituted with the sulfonamide via Lewis acid activation of the quinol leaving group with iron. Rate-limiting oxidation of the iron enolate accounts for the observed linear free energy relationship, as well as the observed product ratios with competing sulfonamides that match reactions of isolated DDQ adducts. The potentially weak coordination of iron to the DDQ could facilitate inner sphere electron transfer, and either this coordination or the formation of the enolate could be prevented by the added coordinating ligand, which significantly decreases the yield (Figure 1).

## Conclusion

This study describes the mechanism of α-amination of ketones mediated by DDQ and catalyzed by iron(III) halide salts. We identified a common intermediate through competition experiments to suggest that nucleophilic substitution occurs after the rate-determining step. Our linear free energy relationship data are consistent with rate-limiting oxidation of an iron enolate, which implicates the quinone-based oxidant directly in the mechanism. This study implies that this mode of activation of carbonyl compounds may be more general for substitution with other nucleophiles. We believe this is the first reported activation of a hydroquinone leaving group with a Lewis acid, which may have implications for other substitution reactions and umpolung approaches for activation of carbonyl compounds.

## Data Availability

The data underlying this study are available in the published article and its Supporting Information.

## Supporting Information Available

Experimental procedures, competition experimental results, and spectroscopic data (PDF)
