# Supplementary material for: Mechanism of Iron-Catalyzed Oxidative α-Amination of Ketones with Sulfonamides
Source: J Org Chem. 2024 Aug 16;89(17):12462–6. doi: 10.1021/acs.joc.4c01401 (PMC11382155; doi:10.1021/acs.joc.4c01401)
Supplement: Supplementary file 1 — jo4c01401_si_001.pdf [file jo4c01401_si_001.pdf]

Mechanism of Iron-Catalyzed Oxidative α-Amination of Ketones with Sulfonamides

Gloria M. Parrales,‡ Nina C. Hollin,‡ Fubin Song, Yangyang Lyu, Anne-Marie O. Martin, and Alexandra E. Strom*

Smith College
Department of Chemistry
100 Green St
Northampton, MA 01106, United States

*Email: astrom@smith.edu

Table of Contents

1. General Information S1
1.1 Reagents and Solvents *S2*
1.2 Experimental Methods *S2*
1.3 Chromatography and Data Analysis *S2*
2. Synthesis of New Amination Products for Comparison in Competition Experiments S2
Compound S1 ..... *S2*
Compound S2[5] *S3*
3. Synthesis of Substrates for Competition Experiments S3
Compound 1c[6] *S3*
Compound 1d *S4*
5. Screening of Oxidants in the Amination Reaction S6
5.1 General Procedure for Table S1 ..... *S6*
6. Optimization of Phosphate and DDQ Adduct Substitution Reactions S6
6.1 General Procedure for Table S2 and Table S3 ..... *S6*
6.2 Competition Optimization Data *S7*
6.3 General Procedure for Competition Experiments with Ketone Electrophiles *S8*
6.4 Competition Experimental Data *S9*
7. Linear Free Energy Relationships S9
7.1 General Procedure for Measuring Relative Rates for LFER Experiments *S9*
7.2 Table of Integration of Products in Competition Reactions *S10*
7.3 Comparison with other Linear Free Energy Parameters *S11*
8. References S12
9. Competition Experiment NMR Line Fitting S13
10. Experimental Spectra for Characterization S15

1. General Information

1.1 Reagents and Solvents

All reagents and solvents were purchased from various commercial sources and used without further purification, including 1,2-dichloroethane (anhydrous, SureSeal, Millipore Sigma), chloroform-D (99.8 atom % D, Cambridge Isotopes), deoxybenzoin (combiblocks), iron tribromide (anhydrous, Strem), benzyl 4-bromophenyl ketone (Acros Organics), benzyl 4-chlorophenyl ketone (TCI America), benzyl 4-fluorophenyl ketone (Matrix Scientific), 4-methylbenzyl phenyl ketone (TCI America), tetrahydrofuran (anhydrous, SureSeal, Millipore Sigma), 1,4-dioxane (anhydrous, Sure Seal, Millipore Sigma), toluene (anhydrous, Sure Seal, Millipore Sigma), *p*-toluenesulfonyl chloride (Thermo Scientific), 2,3-Dichloro-5,6-dicyano-1,4-benzoquinone (Combi-Blocks), and *N*-methyl-*p*-toluenesulfonamide (TCI America). 4-Methylphenyl benzyl ketone,[1] 4-methoxyphenyl benzyl ketone,[2] 4-fluorobenzyl phenyl ketone,[3] and 2-(4-Bromophenyl)-1-phenylethanone,[4] were synthesized via literature procedures.

1.2 Experimental Methods

Iron-catalyzed reactions were assembled in a nitrogen-filled glovebox, tightly sealed, and removed from the glovebox for heating on an aluminum heating block with temperature control. Other reactions were conducted using standard Schlenk techniques under a nitrogen atmosphere to exclude moisture and air, unless otherwise noted.

> ***Caution!*** The procedures in this Supporting Information include reactions in which 1,2-dichloroethane (1,2-DCE) is heated above its boiling point. Risk was minimized by ensuring the volume of the vessel was greater than double the reaction volume and reactions were run on small (0.1-0.2 mmol) scale (0.5-1 mL solvent).

1.3 Chromatography and Data Analysis

Compounds were purified via flash chromatography using either Silicycle siliaFlash P60 silica or Biotage Sfär columns. Thin layer chromatography was performed with Siliaplate silica plates treated with F254 indicator, and visualized with UV light or staining with phosphomolybdic acid stain, or KMNO4 stain, as needed. NMR spectra were recorded on a Bruker 300 MHz or Bruker 500 MHz NMR NMR spectrometer. Chemical shifts are reported in ppm and referenced to chloroform solvent as internal standard. Data is reported as s = singlet, d = doublet, t = triplet, q = quartet, m = multiplet, br = broad, coupling constants are reported in Hz, followed by integration.

2. Synthesis of New Amination Products for Comparison in Competition Experiments

Compound S1

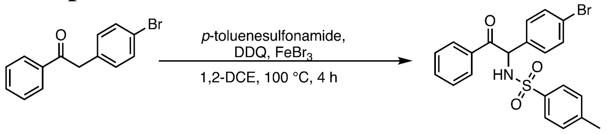

To an oven-dried 4 mL glass vial in a nitrogen-filled glovebox, 2-(4-Bromobenzyl)-1-phenylethanone (0.100 mmol, 1.00 equiv), *p*-toluenesulfonamide (0.300 mmol, 3.00 equiv), DDQ (27.3 mg, 0.120 mmol, 1.20 equiv), iron (III) bromide (5.9 mg, 0.0200 mmol, 0.200 equiv), and an oven-dried stir bar were added. 1,2-DCE (0.5 mL, 0.20 M, anhydrous) was added to the vial, and the vial was sealed with a PTFE-lined cap, removed from the glovebox, and heated to 100 °C in an aluminum heating block for 4 hours. The reaction mixture was allowed to cool to room temperature and then opened to air, and 1 mL of sat. $NH_4Cl_{(aq)}$ was added. The aqueous solution was extracted with DCM until the organic phase was clear (5-10 mL), and the combined organic layers were filtered through a pad of silica, washing with 10% MeOH in DCM (v/v) (20 mL). The crude material was concentrated *in vacuo* and purified by column chromatography, eluting with hexanes/ethyl acetate (0-40%) to afford 13 mg (29% yield) of compound **S1** as a yellow solid. ^{1}H NMR ($CDCl_3$, 500 MHz): δ 7.78 (m, 2H), 7.54-7.48 (m, 3H), 7.37 (t, *J* = 7.5 Hz, 2H), 7.26 (m, 2H), 7.07 (d, *J* = 10 Hz, 2H), 7.03 (m, 2H), 6.26 (d, *J* = 10 Hz, 1H), 5.96 (d, *J* = 10 Hz, 1H), 2.33 (s, 3H). $^{13}C\{^1H\}$ NMR ($CDCl_3$, 126 MHz): δ 197.0, 136.4, 133.43, 133.36, 131.7, 131.2, 128.7, 128.5, 121.0, 44.8. HRMS (m/z): (ESI) calc'd for $C_{21}H_{18}BrNO_3SNa$ [MNa]+: 466.0083, found: 466.0081

Compound S2[5]

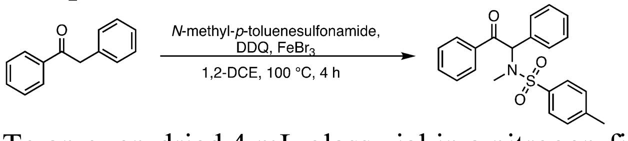

To an oven-dried 4 mL glass vial in a nitrogen-filled glovebox, deoxybenzoin (0.200 mmol, 1.00 equiv), *p*-toluenesulfonamide (0.600 mmol, 3.00 equiv), DDQ (54.5 mg, 0.240 mmol, 1.20 equiv), iron (III) bromide (11.8 mg, 0.0400 mmol, 0.200 equiv), and an oven-dried stir bar were added. 1,2-DCE (0.5 mL, 0.20 M, anhydrous) was added to the vial, and the vial was sealed with a PTFE-lined cap, removed from the glovebox, and heated to 100 °C in an aluminum heating block for 4 hours. The reaction mixture was allowed to cool to room temperature and then opened to air, and 1 mL of sat. $NH_4Cl_{(aq)}$ was added. The aqueous solution was extracted with DCM until the organic phase was clear (5-10 mL), and the combined organic layers were filtered through a pad of silica, washing with 10% MeOH in DCM (v/v) (20 mL). The crude material was concentrated *in vacuo* and purified by column chromatography, eluting with hexanes/ethyl acetate (0-40%) to afford 37.9 mg (52% yield) of Compound **S2** as a white solid.
^{1}H NMR (CDCl3, 300 MHz): δ 7.80- 7.77 (m, 2H), 7.65- 7.61 (m, 2H), 7.54- 7.48 (m, 1H), 7.39- 7.35 (m, 2H), 7.34- 7.30 (m, 3H), 7.25- 7.21 (m, 4H), 6.80 (s, 1H), 2.81 (s, 3H), 2.41 (s, 3H). $^{13}C\{^1H\}$ NMR ($CDCl_3$, 75 MHz): δ 196.9, 143.3, 136.5, 135.4, 134.2, 133.5, 129.8, 129.5, 129.1, 128.8, 128.7, 128.6, 127.3, 64.4, 31.5, 21.5.

3. Synthesis of Substrates for Competition Experiments

Compound 1c[6]

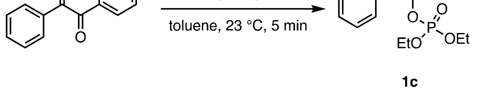

1c

To an oven-dried 10 mL round-bottom flask equipped with a magnetic stir bar, under an atmosphere of N_2 was added benzil (210.0 mg, 1.00 mmol, 1.00 equiv) and anhydrous toluene (5 mL). Triethyl phosphite (0.21 mL, 1.20 mmol, 1.20 equiv) and acetic acid (0.28 mL, 5.00 mmol, 5.00 equiv) were added simultaneously to the stirred solution by syringe. The reaction stirred for five minutes at room temperature and was quenched with aqueous $NaHCO_3$ (2 mL). The reaction mixture was transferred to a 125 mL separatory funnel and extracted with ethyl acetate (25 mL). The aqueous phase was collected and washed with ethyl acetate (3 x 15 mL). The organic layers were combined, dried over anhydrous Na_2SO_4, filtered, and concentrated *in vacuo* under reduced pressure. Crude material was dry-loaded onto celite and washed with 10% ethyl acetate/hexanes (400 mL), 50% ethyl acetate/hexanes (200 mL) and ethyl acetate (200 mL) through a pad of silica loaded onto a glass frit. The product was isolated in the wash of 50% ethyl acetate/hexanes, which was purified further by automated flash column chromatography (15-100% EA/hexanes) to yield **1c** as a translucent oil (253.1 mg, 0.726 mmol, 72.8 %). ^{1}H NMR ($CDCl_3$, 300 MHz): δ 7.93- 7.91 (m, 2H), 7.52- 7.46 (m, 3H), 7.42- 7.32 (m, 5H), 6.63 (d, J= 8.0 Hz, 1H), 4.25- 4.15 (m, 2H), 3.90 (dtd, J=14.5, 7.2, 4.7 Hz, 2H), 1.25 (t, J= 7.1 Hz, 3H), 1.13 (td, J= 7.1, 1.1 Hz, 3H). ^{13}C{^{1}H} NMR ($CDCl_3$, 126 MHz): δ 193.6 (,d, *J* = 4.6 Hz), 134.8 (d, *J* = 5.4 Hz), 134.3, 133.5, 129.3, 129.0, 128.9, 128.6, 128.0, 80.1 (d, *J* = 4.8 Hz), 63.9 (d, *J* = 6.3 Hz), 63.6 (d, *J* = 5.8 Hz), 16.0 (d, *J* = 7.0 Hz), 15.8 (d, *J* = 7.1 Hz).

Compound 1d

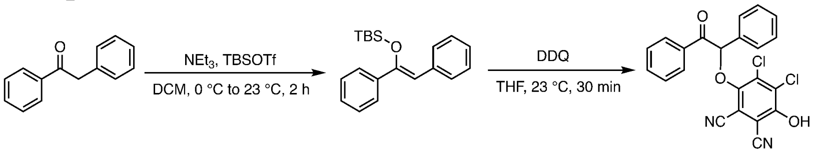

S3 1d

Compound S3. An oven-dried 10 mL round-bottom flask was charged with a stir bar and sparged with N_2 for ten minutes. Deoxybenzoin (196.2 mg, 1.00 mmol, 1.00 equiv) was added to the flask and dissolved in anhydrous DCM (10 mL, 0.1 M) with stirring. To the stirred solution was added triethylamine (0.17 mL, 1.20 mmol, 1.20 equiv), followed by *t*-butyldimethylsilyl trifluoromethanesulfonate (0.28 mL, 1.10 mmol, 1.10 equiv) at 0 °C. The reaction mixture was allowed to warm while stirring for 2 h with the ice bath removed. The reaction was quenched with saturated $NH_4Cl_{(aq)}$ solution (2 mL) and the layers were separated. The aqueous layer was extracted with diethyl ether (2 x 5 mL). The organic layers were combined and dried over anhydrous sodium sulfate, filtered, and concentrated by rotary evaporation under reduced pressure. The crude product was purified by automated flash column chromatography on silica (100% hexanes) to yield **S3** (184.9 mg, 0.600 mmol, 59.5 %) as a colorless oil. ^{1}H NMR ($CDCl_3$, 500 MHz): δ 7.64 (m, 2H), δ 7.59 (m, 2H), δ 7.17 (m, 1H), 6.11 (s, 1H), δ 0.97 (s, 9H), δ -0.22 (s, 6H). ^{13}C{^{1}H} NMR ($CDCl_3$, 126 MHz): δ 151.1, 140.2, 136.6, 128.9, 128.1, 127.9, 126.3, 126.0, 110.9, 26.0, 18.3, -3.9.

Compound 1d. Compound **S3** (377.0 mg, 1.214 mmol, 1.00 equiv) was added to a 50 mL round-bottom flask and dissolved in anhydrous THF (12.1 mL, 0.10 M) with stirring. 2,3-Dichloro-5,6-dicyano-1,4-benzoquinone (227.0 mg, 1.336 mmol, 1.10 equiv) was added to the reaction vessel. The vial was capped and the reaction mixture was allowed to stir at room temperature for one hour. The reaction was stopped and THF was removed *in vacuo* under reduced pressure to yield crude product as a green oil. The reaction mixture was purified using automated flash column chromatography on silica gel (0-40% EtOAc/hexanes) to afford pure **1d** as a white powder (308.5 mg, 0.729 mmol, 60.5 %). ^{1}H NMR (CD_3CN, 500 MHz): δ 7.91-7.88 (m, 2H), 7.65- 7.53 (m, 1H), 7.47- 7.45 (m, 2H), 7.44- 7.40 (m, 2H), 7.38- 7.33 (m, 3H), 6.85 (s,

1H), 2.30- 2.10 (br s, 1H). $^{13}C\{^1H\}$ NMR (CD_3CN, 126 MHz): δ 193.8, 154.2, 151.3, 135.2, 135.1, 134.9, 134.6, 131.0, 130.26, 130.25, 129.9, 129.6, 129.2, 113.7, 113.5, 110.6, 102.9, 87.6. HRMS (m/z): (ESI) calc'd for $C_{22}H_{12}Cl_2N_2O_3Na$ [MNa]+: 445.0117, found: 445.0105

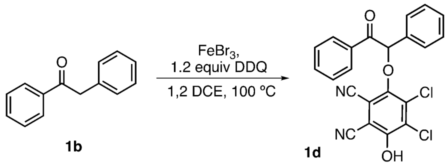

Compound 1d (alternate route, as shown above and in Scheme 2).
To an oven-dried 4 mL glass vial equipped with an oven-dried stir bar in a nitrogen-filled glovebox, deoxybenzoin (39.2 mg, 0.200 mmol, 1.00 equiv), DDQ (54.5 mg, 0.240 mmol, 1.20 equiv), and iron (III) bromide (11.8 mg, 0.0400 mmol, 0.200 equiv) were added. 1,2-DCE (1 mL, 0.20 M, anhydrous) was added to the vial, and the vial was sealed with a PTFE-lined cap, removed from the glovebox, and heated to 100 °C in an aluminum heating block for 1 h. The reaction mixture was allowed to cool to room temperature and then opened to air, and 1 mL of sat. $NH_4Cl_{(aq)}$ was added. The aqueous solution was extracted with DCM until the organic phase was clear (5-10 mL), and the combined organic layers were filtered through a pad of silica, washing with DCM (20 mL) (Wash **A**). The silica pad was then flushed with 20% MeOH in EtOAc (v/v) (20 mL) into a separate flask (wash **B**). Ethylene carbonate (8.8 mg, 0.10 mmol, 0.50 equiv) was added as an internal standard to the MeOH/EtOAc filtrate (wash **B**) and the solution was concentrated *in vacuo*. The mixture was analyzed by ^{1}H NMR (CD_3CN), and authentic **1d** was added to confirm the identity of the observed species (See Figure S1).

Figure S1. ^{1}H NMR analysis of *in situ* generated **1d**.

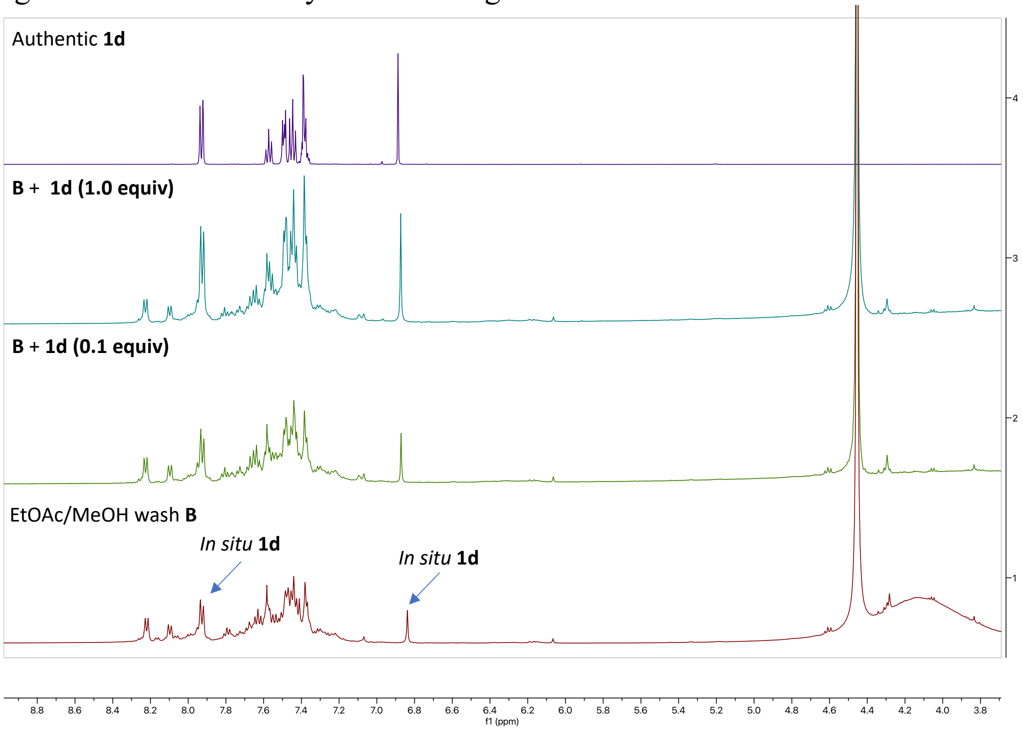

5. Screening of Oxidants in the Amination Reaction

5.1 General Procedure for Table S1

To an oven-dried vial in the glovebox was added 1-(4-fluorophenyl)-2-phenylethanone (42.8 mg, 0.200 mmol, 1.00 equiv), DDQ (54.5 mg, 0.240 mmol, 1.20 equiv), *p*-toluenesulfonamide (103 mg, 0.600 mmol, 3.00 equiv), iron(III) bromide (11.8 mg, 0.0400 mmol, 0.200 equiv) and an oven-dried stir bar. 1,2-DCE (1.0 mL, 0.20 M, anhydrous) was added, and the reaction was sealed with a PTFE-lined cap, removed from the glovebox, and heated to 100 °C for four hours in an aluminum heating block. The reaction was allowed to cool to room temperature, then opened to air and 1 mL sat $NH_4Cl_{(aq)}$ was added. The aqueous solution was extracted with DCM until the organic phase was clear, and the combined organic layers were filtered through a pad of silica, washing with 20% MeOH in DCM (10 mL). Ethylene carbonate (8.8 mg, 0.10 mmol, 0.5 equiv) was added and the solvent was removed in vacuo. The crude solid was dissolved in $CDCl_3$ (0.5 mL) and a portion of the $CDCl_3$ solution was diluted further with $CDCl_3$ for ^{1}H NMR analysis. ^{1}H NMR is taken with a delay of 6 seconds and 32 scans on a 500 MHz NMR.

Table S1. Screening of additional oxidants

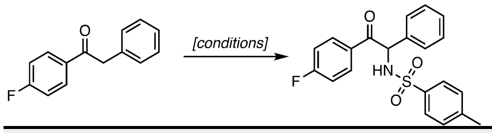

Entry	Oxidant (equiv)	yield (%)[a]
1	DDQ (1.2)[7]	77
2	benzoquinone (1.2)[7]	6
3	*p*-chloranil (1.2)[7]	37
4	*o*-chloranil (1.2)[7]	23
5	PIDA (1.2)[7]	5
6	*t*-butyl peroxybenzoate (1.2)	16
7	oxone (1.2)	ND
8	Dess-Martin Periodinane (1.2)[7]	ND
9	$NaIO_4$ (1.2)	ND
10	dicumylperoxide	ND

[a] ND = not detected.

6. Optimization of Phosphate and DDQ Adduct Substitution Reactions.

6.1 General Procedure for Table S2 and Table S3

To an oven-dried 4 mL glass vial inside the glovebox was added 2-Diethylphosphoryloxy-1,2-diphenylethanone (34.8 mg, 0.100 mmol, 1.00 equiv), sulfonamide (0.300 mmol, 3.00 equiv), oxidant (0.120 mmol, 1.20 equiv), catalyst (0.0200 mmol, 0.200 equiv), and a micro stir bar. The reagents were dissolved in anhydrous 1,2-DCE (0.5 mL, 0.20 M). The reaction vessel was tightly sealed, removed from the glovebox, and stirred at 100 °C for four hours in an aluminum heating block. The reaction was allowed to cool to room temperature, opened to air, and quenched with saturated $NH_4Cl_{(aq)}$ solution (0.5 mL). The aqueous phase was extracted repeatedly with DCM until the organic layer was colorless. The organic layers were transferred to a pad of silica on a glass frit and washed with 10% MeOH in DCM (v/v). Ethylene carbonate was added (8.8 mg, 0.100 mmol) and the solvent was removed by rotary evaporation under reduced pressure. The crude material was dissolved in $CDCl_3$ (0.5 mL). A portion of the crude solution was transferred to an NMR tube and diluted further with $CDCl_3$ for analysis by ^{1}H NMR spectroscopy. ^{1}H NMR is taken with a delay of 6 seconds and 32 scans on a 500 MHz NMR.

6.2 Competition Optimization Data

Table S2. Optimization of α-Amination with **1c**

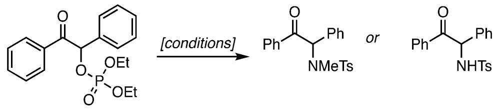

1c **2b** **2c**

Entry	Catalyst (mol %)	Oxidant (equiv)	Time (h)	**Product**	Yield (%)
1	$FeBr_3$ (20)	-	4	**2c**	4.0
2	$FeBr_3$ (20)	-	24	**2c**	4.0
3	$FeBr_3$ (100)	-	4	**2c**	8.0
4	$FeBr_3$ (20)	DDQ (1.2)	4	**2c**	< 4.0
5	$AlCl_3$ (20)	-	4	**2c**	ND
6	$AlCl_3$ (100)	-	4	**2c**	< 4.0
7	$ZnCl_2$ (20)	-	4	**2c**	ND
8	$ZnBr_2$ (20)	-	4	**2c**	ND
9	$SnCl_2$ (20)	-	4	**2c**	< 4.0
10	TTIP (20)	-	4	**2c**	ND
11	$TiCl_4$ (20)	-	4	**2c**	ND
12	TMSOTf (20)	-	4	**2c**	64.0
13	TMSOTf (20)	-	4	**2b**	64.0

Liquid phase catalysts were added outside the glovebox to the reaction vessel sealed with a PTFE-lined puncturable screw cap using a 1000 μL glass microsyringe under vigorously bubbling N_2. The cap was quickly interchanged for a PTFE-lined melamine resin cap following catalyst addition prior to heating. ND = not detected.

Table S3. Optimization of α-Amination with **1d**

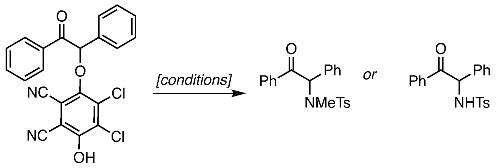

1d **2b** **2c**

Entry	Oxidant (equiv)	Time (h)	**Product**	Yield (%)
1	-	4	**2c**	12.7
2	-	24	**2b**	37.4
3	DDQ (1.2)	4	**2c**	25.9
4	DDQ (1.2)	4	**2b**	50.0

6.3 General Procedure for Competition Experiments with Ketone Electrophiles

A typical procedure for the setup and execution of competition experiments between *p*-toluene sulfonamide and *N*-methyl-*p*-toluene sulfonamide is described. A 1:1 stock solution of both sulfonamides in anhydrous MeOH (Sigma-Aldrich, ≥ 99.8%) was prepared at 0.75 M. *N*-methyl-*p*-toluene sulfonamide (0.6947 g, 3.750 mmol) and *p*-toluene sulfonamide (0.6420 g, 3.750 mmol) were weighed and combined in a 40 mL glass scintillation vial. The contents of the vial were dissolved in MeOH (6.0 mL) and swirled vigorously by hand for two minutes. A glass pipette was used to transfer the solution into a 10 mL volumetric flask. The scintillation vial was washed three times with MeOH (1 mL) and each wash was added to the flask. The volumetric flask was topped with MeOH to 10.0 mL. The solution was distributed among twelve 4 mL 1 dram vials (800 µL/vial) using a 1000 µL glass microsyringe. The vials were concentrated *in vacuo* until half of the solution remained, diluted with anhydrous toluene (0.4 mL), and concentrated until almost complete dryness at 300 bpm, 24 °C. The vials were transferred to the glovebox antechamber, where the vials were left to continue drying over a period of 72-96 hours.

To a vial inside the glovebox containing 1:1 *p*-toluene sulfonamide (0.300 mmol, 1.50 equiv): *N*-methyl-*p*-toluene sulfonamide (0.300 mmol, 1.50 equiv), was added ketone (0.200 mmol, 1.00 equiv), DDQ (54.5 mg, 0.240 mmol, 1.20 equiv), catalyst (0.0400 mmol, 0.200 equiv), and a micro stir-bar. Anhydrous 1,2-DCE (1.0 mL, 0.20 M) was added to the vial via syringe. The reaction was sealed with a PTFE-lined melamine resin cap, removed from the glovebox, and heated at 100 °C in an aluminum heating block. The reaction was cooled in a bath of dry ice/acetone, opened to air, and quenched with saturated $NH_4Cl_{(aq)}$ solution (1 mL). The aqueous layer was extracted with DCM until the organic phase was clear. The combined organic layers were filtered through a bed of silica on a glass frit with 10% MeOH:DCM (v/v) into a 250 mL round-bottom flask. Ethylene carbonate (8.8 mg, 0.100 mmol) was added and the solvent was removed by rotary evaporation under reduced pressure. The concentrated material was dissolved in $CDCl_3$ (0.5 mL) and a portion of the solution was diluted further with $CDCl_3$ for analysis by ^1H NMR spectroscopy.

6.4 Competition Experimental Data

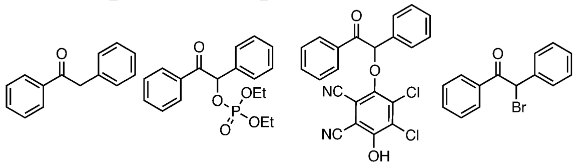

1b **1c** **1d** **1e**

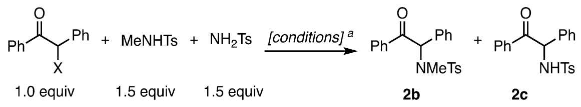

Table S4.

Entry	Precursor	Catalyst (mol %)	Oxidant (equiv)	Time (min)	Product yield (**2b** %, **2c** %)	Product Ratio (**2b**:**2c**)	Product ratio (average over *n* runs, **2b**:**2c**)
1	**1b**	$FeBr_3$ (20)	DDQ (1.2)	10	24.8, 14.3	1.00 : 0.57	
2	**1b**	$FeBr_3$ (20)	DDQ (1.2)	10	24.2, 13.8	1.00 : 0.57	1.00 : 0.57
3	**1b**	$FeBr_3$ (20)	DDQ (1.2)	10	23.8, 13.9	1.00 : 0.58	
4	**1b**	$FeBr_3$ (20)	DDQ (1.2)	6	5.2, 2.6	1.00 : 0.50	
5	**1b**	$FeBr_3$ (20)	DDQ (1.2)	6	2.0, 1.1	1.00 : 0.54	1.00 : 0.54
6	**1b**	$FeBr_3$ (20)	DDQ (1.2)	6	14.7, 8.4	1.00 : 0.57	
7	**1c**	TMSOTf (20)	-	10	16.1, 4.8	1.00 : 0.92	
8	**1c**	$FeBr_3$ (20)	-	6	11.8, 10.5	1.00 : 0.89	1.00 : 0.91
9	**1c**	$FeBr_3$ (20)	-	5	11.0, 10.3	1.00 : 0.94	
10	**1c**	$FeBr_3$ (20)	-	3	9.0, 8.1	1.00 : 0.90	
11	**1d**	$FeBr_3$ (20)	DDQ (1.2)	10	20.1, 12.2	1.00 : 0.59	
12	**1d**	$FeBr_3$ (20)	DDQ (1.2)	6	3.0, 1.7	1.00 : 0.56	1.00 : 0.57
13	**1d**	$FeBr_3$ (20)	DDQ (1.2)	6	2.9, 1.7	1.00 : 0.58	
14	**1c**	TMSOTf (80)	DDQ (1.2)	6	30.1, 28.4	1.00 : 0.94	-
15	**1e**	$AgPF_6$ (1.2)	-	6	43.4, 45.4	1.00 : 1.04	-

7. Linear Free Energy Relationships

7.1 General Procedure for Measuring Relative Rates for LFER Experiments

One of the following methods was employed to dose vials with 0.5 equiv of deoxybenzoin and 0.5 equiv substituted ketone:

A) 0.5 mmol each of deoxybenzoin and ketone were dissolved in 2 mL DCE. 0.4 mL of this solution was pipetted using a syringe with plunger into a 4 mL vial.

B) 0.1 mmol ketone was added into preweighed 4 mL vials. Then 0.2 mL of a 0.5 M solution of deoxybenzoin was added to each vial.

C) 0.5 mmol each of deoxybenzoin and ketone were dissolved in 5 mL DCM. 1 mL of this solution was pipetted using a syringe with plunger into a 4 mL vial.

D) 0.1 mmol of each ketone was weighed into each vial.

In all cases, the solutions/suspensions containing 0.1 mmol of each ketone were concentrated to dryness and transferred to a glove box. Then to each vial *p*-toluenesulfonamide (103 mg, 0.6 mmol, 3 equiv), DDQ (54.5 mg, 0.24 mmol, 1.2 equiv), $FeBr_3$ (11.8 mg, 0.04 mmol, 0.2 equiv), and an oven-dried stir bar were added. 1,2-DCE (1.0 mL, 0.20 M, anhydrous) was added to each vial, and the vials were sealed with PTFE-lined caps, removed from the glovebox, and heated to 100 ℃ in an aluminum heating block for 10 minutes. The vials were immediately immersed in a cold-water bath to cool to room temperature and then opened to air, and 1 mL of sat. $NH_4Cl_{(aq)}$ was added. The aqueous solution was extracted with DCM until the organic phase was clear (5-10 mL), and the combined organic layers were filtered through a pad of silica, washing with 10% MeOH in DCM (50 mL) (v/v). Ethylene carbonate (0.2 mL of a 0.25 M solution or 4.4 mg) was added, and the solvent was removed in vacuo. The crude solid was dissolved in $CDCl_3$ (4 mL), and a portion of the $CDCl_3$ solution was used for ^{1}H NMR analysis. ^{1}H NMR is taken with a delay of 6 seconds and 32 scans on a 500 MHz NMR. The MestreNova Line Fit tool is used to determine the integrations of the α-proton peaks or tosyl methyl peaks in each product (*vide infra*).

7.2 Table of Integration of Products in Competition Reactions

Table S5. Data summary of competition experiments for phenyl substituents.

		Phenyl Substituent – Peak Area (integration)			
Entry	Product	OMe	Me	F	Br
1	**H**	14884.427	10128.417	16177.74	20593.524
2	**H**	16232.511	10798.27	11894.126	19201.225
3	**H**	16994.92	12777.426	11153.407	22879.341
4	**X**	28085.518	15581.336	11766.831	12981.969
5	**X**	30182.242	16699.497	8863.081	12438.562
6	**X**	31952.199	17858.18	8035.819	14604.108
7	**X/H (avg)**	1.87545968	1.49417028	0.73099756	0.6388337
8	**Std. dev.**	0.01434338	0.08370036	0.01274021	0.00871661
9	**Log(X/H)**	0.27310773	0.17440009	-0.1360841	-0.1946122
15	**Integration notes**	α-C-H	α-C-H	α-C-H	α-C-H

=

Table S6. Data summary of competition experiments for benzyl substituents.

		Benzyl Substituent – Peak Area (integration)			
Entry	Product	OMe	Me	F	Br
1	**H**	18911.883	22714.391	4275	43773.832
2	**H**	23671.218	25367.114	14395.224	
3	**H**	24180.188	12138.493	9252.247	
4	**X**	27718.166	20773.737	3026.124	28762.264
5	**X**	34990.235	22048.82	9978.23	
6	**X**	36227.567	11739.552	6288.731	
7	**X/H (avg)**	1.48068599	0.91696205	0.69357517	0.65706525
8	**Std. dev.**	0.01643699	0.0490166	0.01408831	
9	**Log(X/H)**	0.17046297	-0.0376486	-0.1589065	-0.1823915
15	**Integration notes**	α-C-H	methyl peaks	α-C-H	methyl peaks

7.3 Comparison with other Linear Free Energy Parameters

Chart S1. Log (X/H) compared with Creary[8,9] (radical) parameter for benzyl substitution

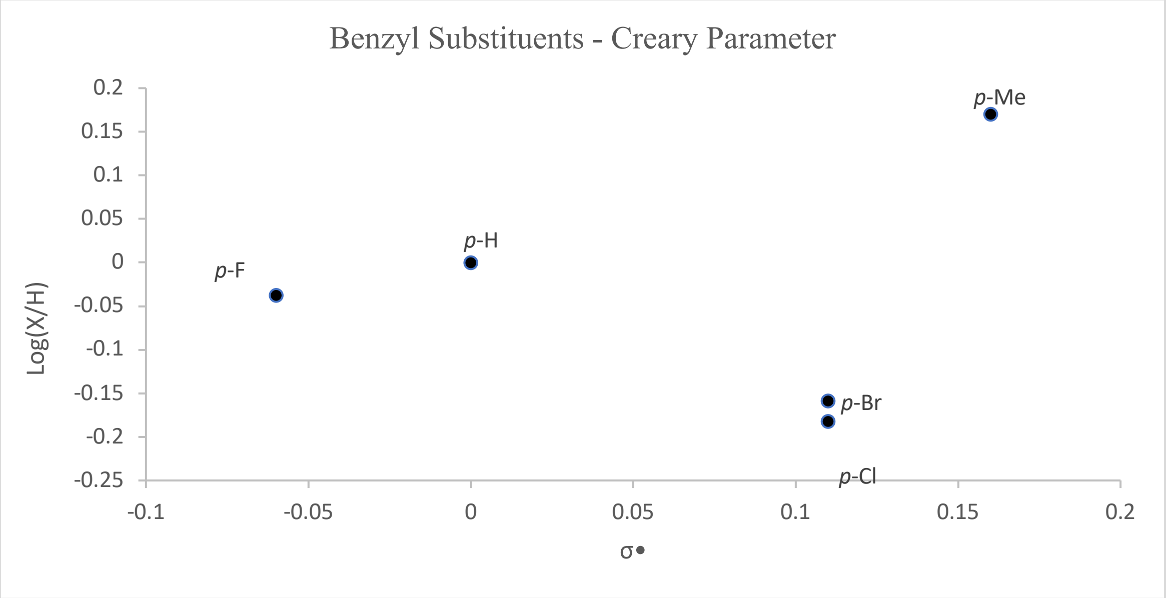

Chart S2. Log (X/H) compared with Creary (radical) parameter for phenyl substitution

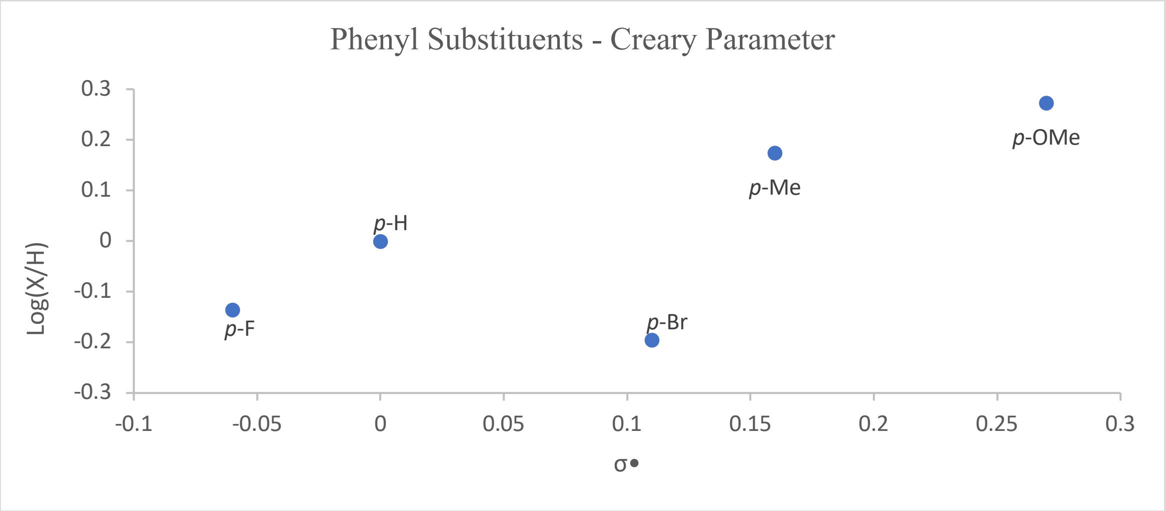

8. References

(1) Lütjens, H.; Zickgraf, A.; Figler, H.; Linden, J.; Olsson, R. A.; Scammells, P. J. 2-Amino-3-Benzoylthiophene Allosteric Enhancers of A1 Adenosine Agonist Binding: New 3, 4-, and 5-Modifications. *J. Med. Chem.* **2003**, *46* (10), 1870–1877. https://doi.org/10.1021/jm020295m.

(2) Jung, J.-C.; Lee, J.-H.; Oh, S.; Lee, J.-G.; Park, O.-S. Synthesis and Antitumor Activity of 4-Hydroxycoumarin Derivatives. *Bioorganic & Medicinal Chemistry Letters* **2004**, *14* (22), 5527–5531. https://doi.org/10.1016/j.bmcl.2004.09.009.

(3) Cao, C.; Wang, L.; Cai, Z.; Zhang, L.; Guo, J.; Pang, G.; Shi, Y. Palladium-Catalyzed α-Ketone Arylation under Mild Conditions. *European Journal of Organic Chemistry* **2011**, *2011* (8), 1570–1574. https://doi.org/10.1002/ejoc.201001428.

(4) Yang, S.; Dai, C.; Chen, Y.; Jiang, Y.; Shu, S.; Huang, Z.; Zhao, Y. Ruthenium(II)-Catalyzed Cross-Coupling of Benzoyl Formic Acids with Toluenes: Synthesis of 2-Phenylacetophenones. *European Journal of Organic Chemistry* **2021**, *2021* (21), 2955–2961. https://doi.org/10.1002/ejoc.202100432.

(5) Gediya, S. K.; Clarkson, G. J.; Wills, M. Asymmetric Transfer Hydrogenation: Dynamic Kinetic Resolution of α-Amino Ketones. *J. Org. Chem.* **2020**, *85* (17), 11309–11330. https://doi.org/10.1021/acs.joc.0c01438.

(6) Ramanjaneyulu, B. T.; Vidyacharan, S.; Yim, S. J.; Kim, D.-P. Fast-Synthesis of α-Phosphonyloxy Ketones as Drug Scaffolds in a Capillary Microreactor. *European Journal of Organic Chemistry* **2019**, *2019* (47), 7730–7734. https://doi.org/10.1002/ejoc.201901655.

(7) Song, F.; Park, S. H.; Wu, C.; Strom, A. E. Iron-Catalyzed Oxidative α-Amination of Ketones with Primary and Secondary Sulfonamides. *J. Org. Chem.* **2023**, *88* (5), 3353–3358. https://doi.org/10.1021/acs.joc.3c00210.

(8) Creary, X.; Engel, P. S.; Kavaluskas, N.; Pan, L.; Wolf, A. Methylenecyclopropane Rearrangement as a Probe for Free Radical Substituent Effects. σ • Values for Potent

Radical-Stabilizing Nitrogen-Containing Substituents. *J. Org. Chem.* **1999**, *64* (15), 5634–5643. https://doi.org/10.1021/jo990732d.
(9) Creary, X. Super Radical Stabilizers. *Acc. Chem. Res.* **2006**, *39* (10), 761–771. https://doi.org/10.1021/ar0680724.

9. Competition Experiment NMR Line Fitting

The following are line-fitting reports generated in Mestrenova for competition experiments as summarized in Table S5 and S6.

Competition Experiments: para-Phenyl Substituents:

NMR	Line Fitting Report
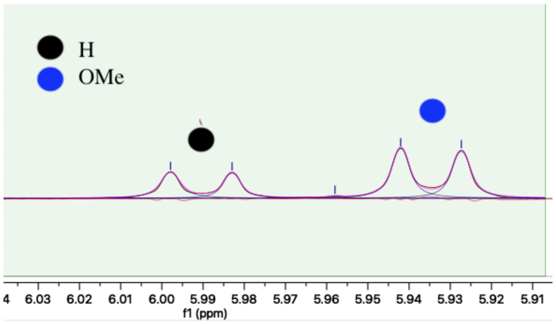	Name: M1<br>From: 5.907 ppm<br>To: 6.053 ppm<br>Residual Error:142<br># ppm Height Width(Hz) L/G Area<br>1 5.9979 307.92 2.82 0.42 8153.617<br>2 5.9830 296.65 2.52 0.73 6730.810<br>3 5.9580 13.65 1.71 1.36 190.229<br>4 5.9420 590.81 2.61 0.45 14450.321<br>5 5.9273 550.54 2.74 0.69 13635.197
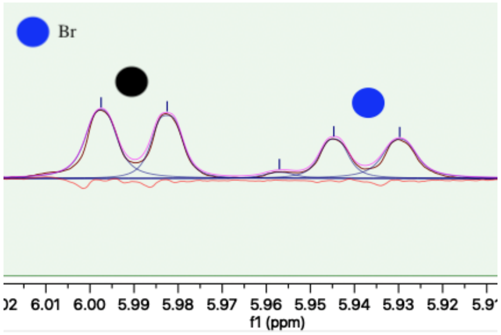	Name: M1<br>From: 5.906 ppm<br>To: 6.062 ppm<br>Residual Error:178<br># ppm Height Width(Hz) L/G Area<br>1 5.9975 331.26 4.10 1.15 11441.808<br>2 5.9825 302.16 4.02 1.82 9151.716<br>3 5.9570 29.12 4.11 1.12 1013.969<br>4 5.9447 189.60 3.75 1.44 5727.693<br>5 5.9297 189.72 4.45 1.03 7254.276

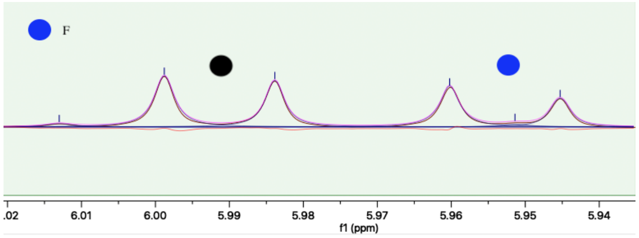

Name: M1
From: 5.935 ppm
To: 6.049 ppm
Residual Error:433

#	ppm	Height	Width(Hz)	L/G	Area
1	6.0130	35.22	2.37	-0.06	837.299
2	5.9988	543.43	1.64	0.10	8742.007
3	5.9839	493.51	1.55	0.17	7435.733
4	5.9602	436.94	1.58	-0.14	6996.335
5	5.9514	28.39	2.50	0.07	701.414
6	5.9453	305.20	1.65	0.38	4770.496

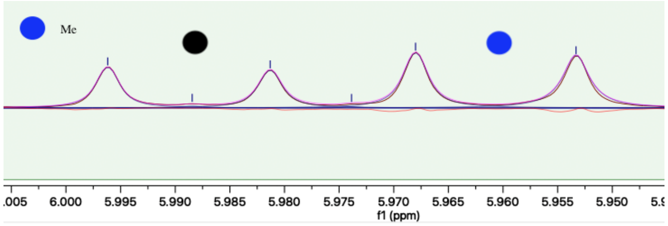

Name: M1
From: 5.940 ppm
To: 6.034 ppm
Residual Error:581

#	ppm	Height	Width(Hz)	L/G	Area
1	5.9962	419.52	1.30	0.37	5159.420
2	5.9884	20.03	1.48	-0.14	301.376
3	5.9813	388.50	1.34	0.29	4968.997
4	5.9739	13.11	1.01	1.19	110.657
5	5.9680	568.87	1.39	0.29	7547.431
6	5.9533	545.40	1.47	-0.05	8033.905

Competition Experiments: para-Benzyl Substituents

NMR	Line Fitting Report

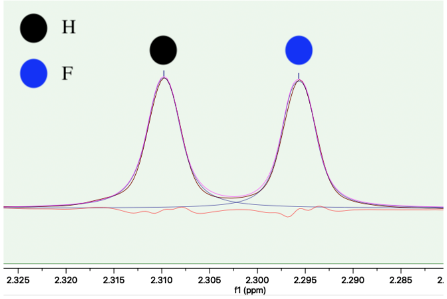

Name: M1
From: 2.280 ppm
To: 2.346 ppm
Residual Error:399

#	ppm	Height	Width(Hz)	L/G	Area
1	2.3098	1242.25	2.06	0.80	22714.391
2	2.2957	1208.66	2.06	1.20	20773.738

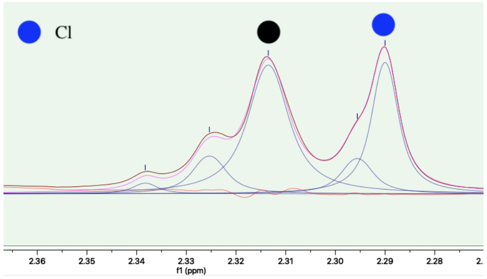

Name: M1
From: 2.268 ppm
To: 2.369 ppm
Residual Error:300

#	ppm	Height	Width(Hz)	L/G	Area
1	2.3383	77.97	3.04	0.31	2260.094
2	2.3254	275.31	4.06	0.54	10326.316
3	2.3135	936.97	4.89	0.31	43773.833
4	2.2956	257.14	3.82	0.48	9153.819
5	2.2900	956.18	3.22	0.46	28762.264

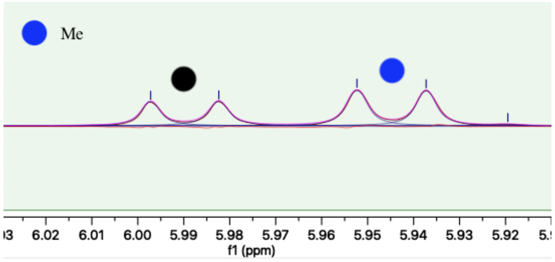

Name: M1
From: 5.900 ppm
To: 6.038 ppm
Residual Error:192

#	ppm	Height	Width(Hz)	L/G	Area
1	5.9972	379.40	2.52	0.41	9007.945
2	5.9824	387.27	2.66	0.26	9903.938
3	5.9523	552.24	2.80	0.61	14151.856
4	5.9373	547.91	2.67	0.52	13566.310
5	5.9195	19.74	3.72	0.44	688.808

10. Experimental Spectra for Characterization

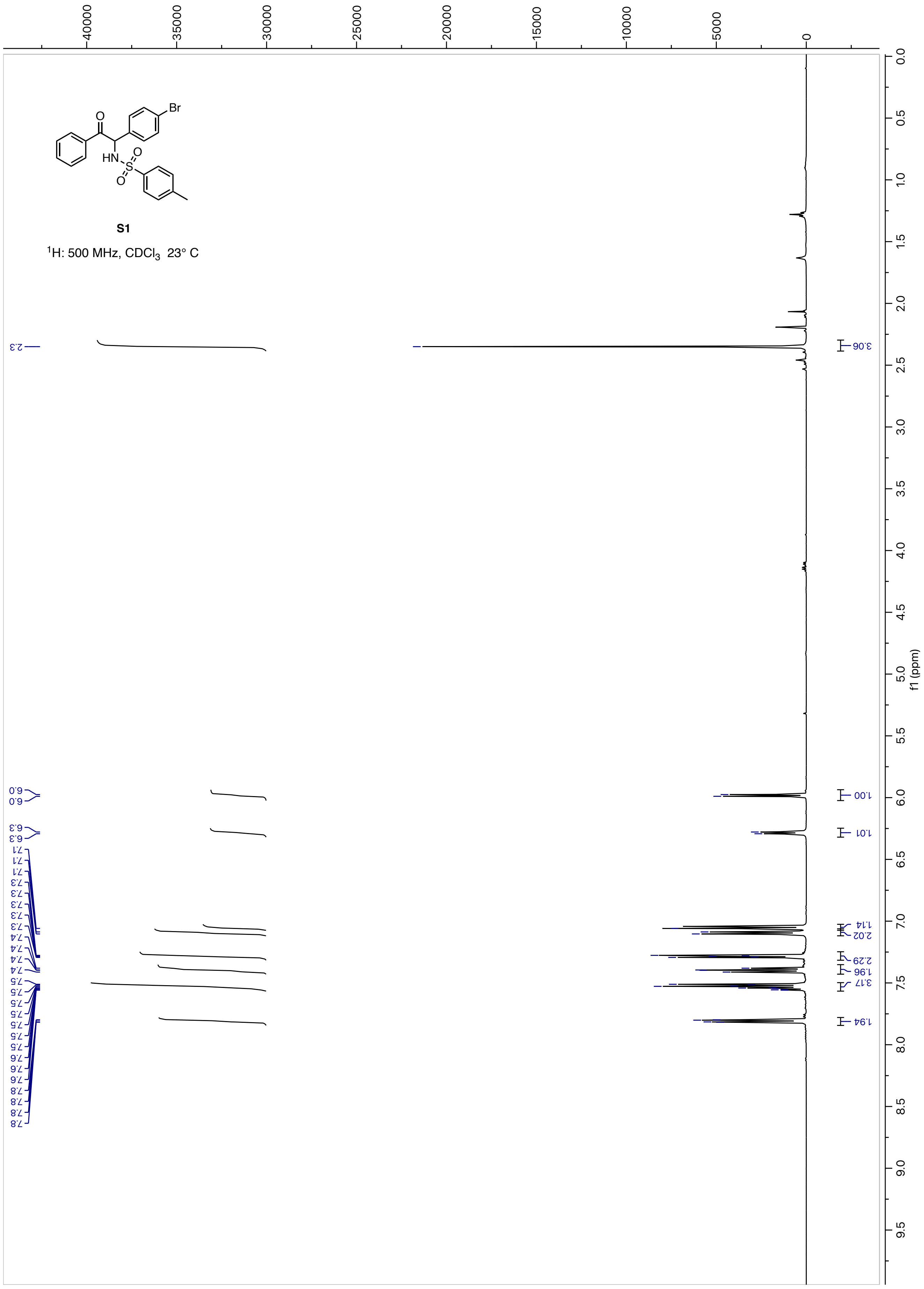
S1
1H: 500 MHz, CDCl3 23° C
f1 (ppm)

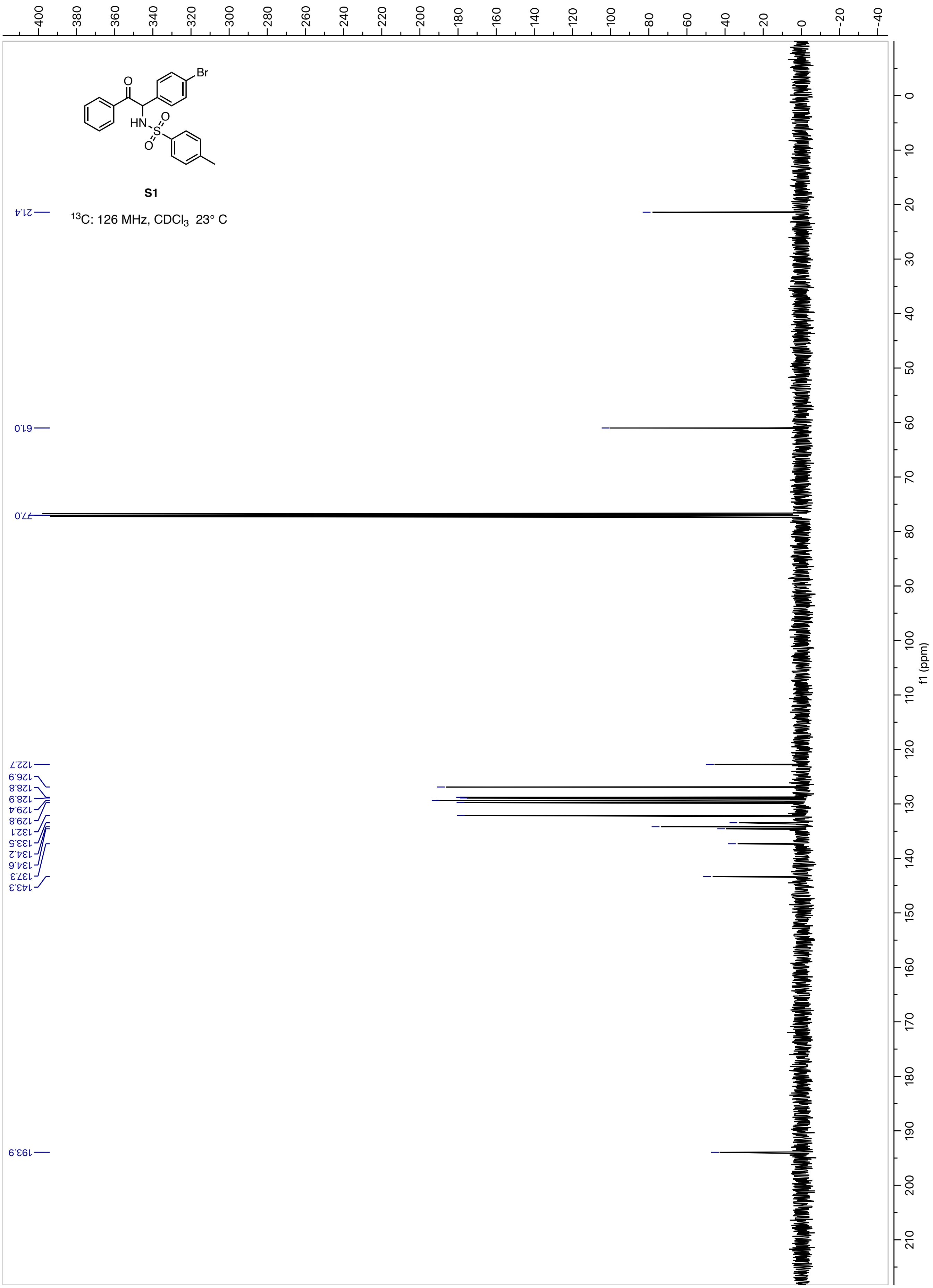

Br
O
HN
S
O
O
S1
^{13}C: 126 MHz, $CDCl_3$ 23° C
21.4
61.0
77.0
122.7
126.9
128.8
128.9
129.4
129.8
132.1
133.5
134.2
134.6
137.3
143.3
193.9
f1 (ppm)

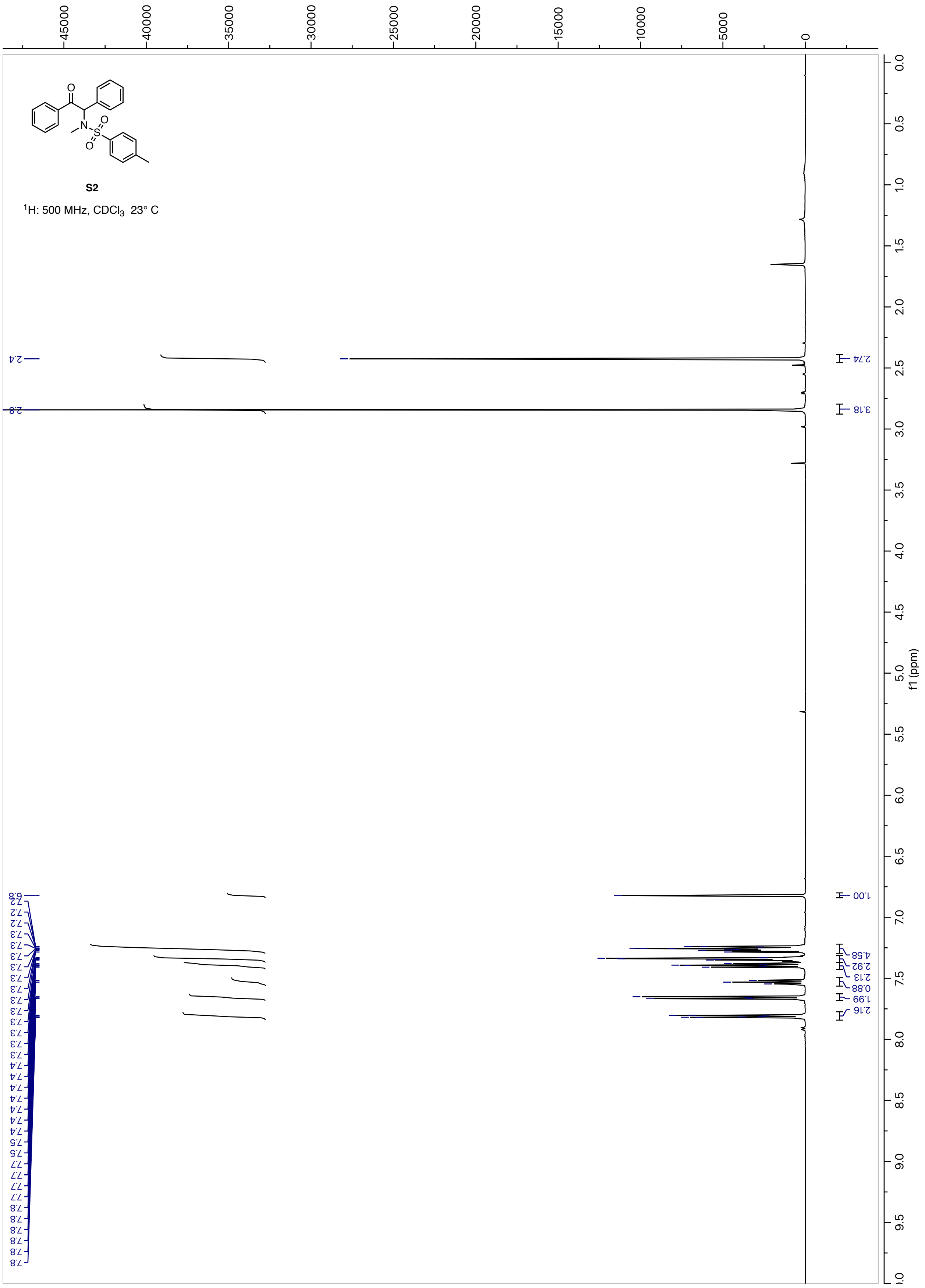
S2
1H: 500 MHz, CDCl3 23° C
f1 (ppm)

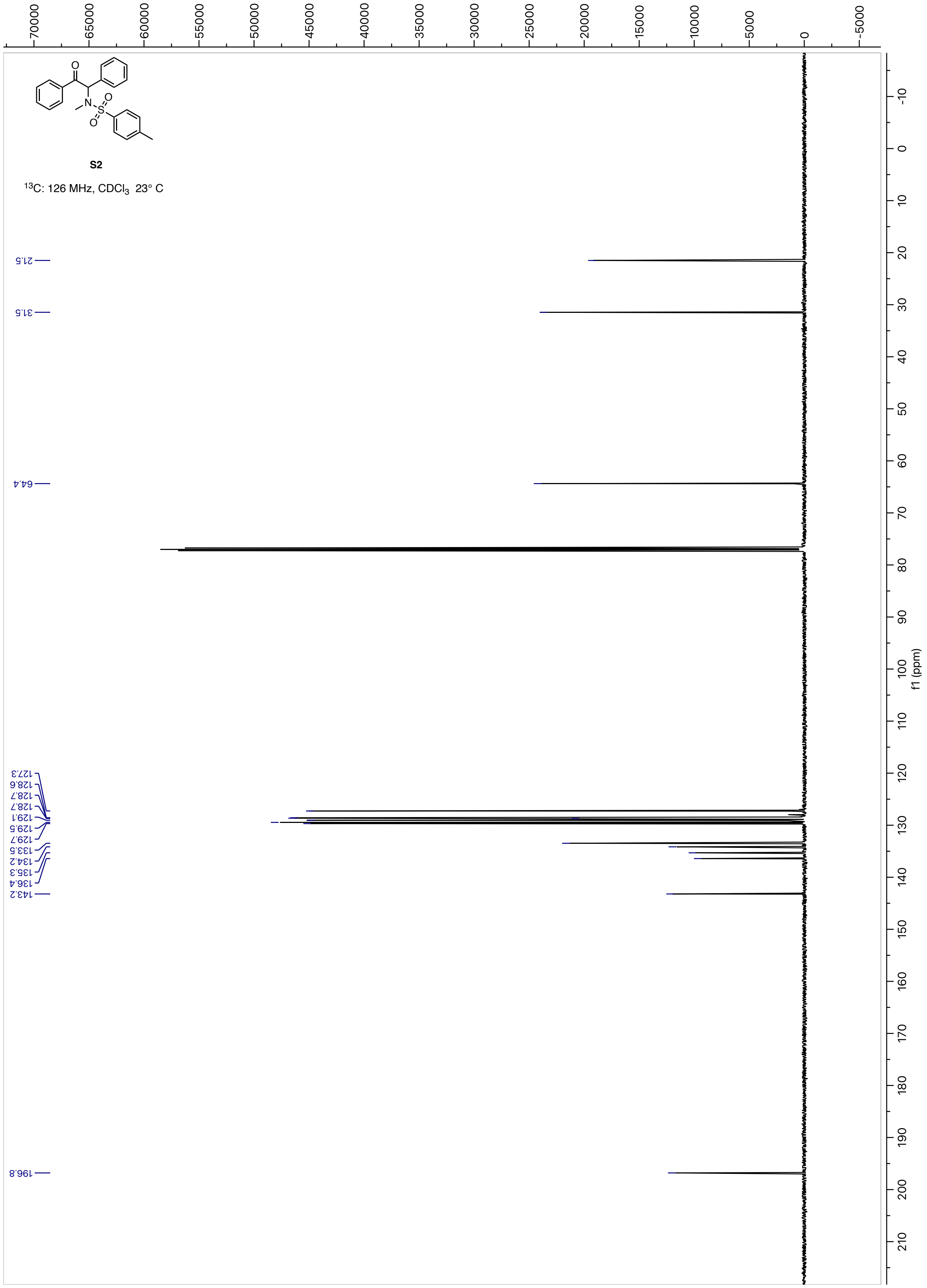
S2
^{13}C: 126 MHz, CDCl$_3$ 23° C
21.5
31.5
64.4
127.3
128.6
128.7
128.7
129.1
129.5
129.7
133.5
134.2
135.3
136.4
143.2
196.8
f1 (ppm)

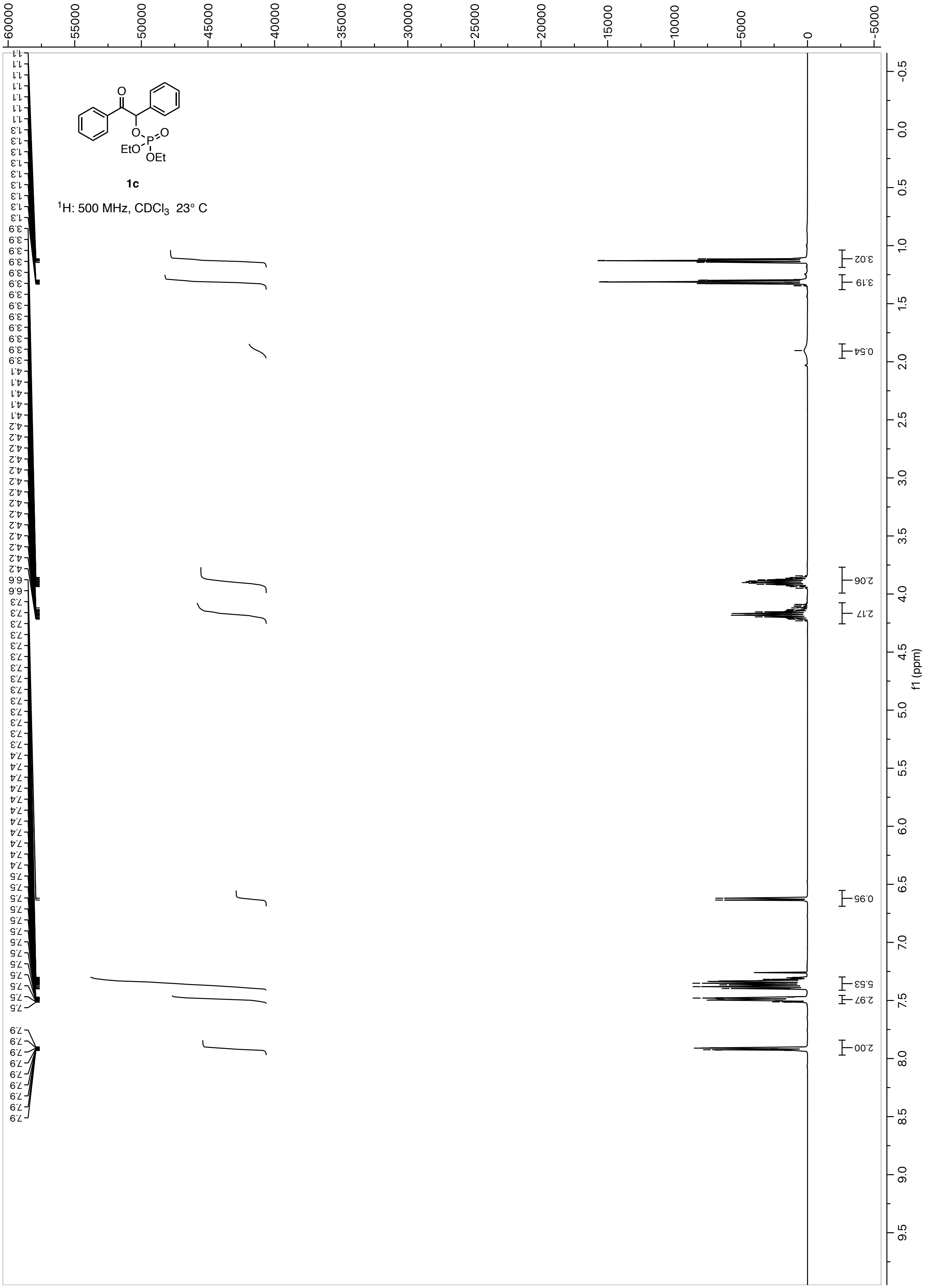

O
EtO
OEt
1c
1H: 500 MHz, CDCl3 23° C
f1 (ppm)
3.02
3.19
0.54
2.06
2.17
0.95
5.53
2.97
2.00

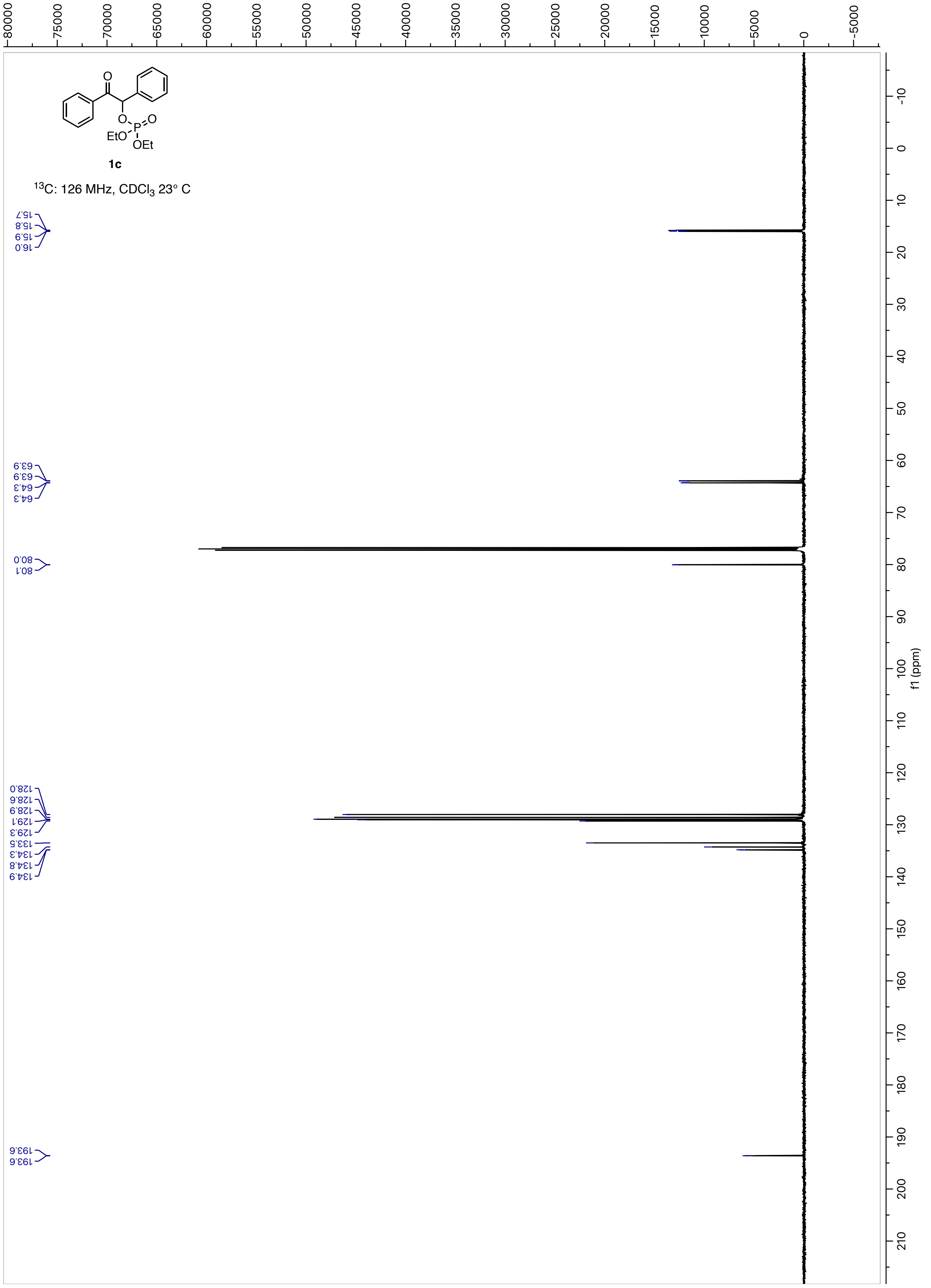
1c
13C: 126 MHz, CDCl3 23° C
15.7
15.8
15.9
16.0
63.9
63.9
64.3
64.3
80.0
80.1
128.0
128.6
128.9
129.1
129.3
133.5
134.3
134.8
134.9
193.6
193.6
f1 (ppm)

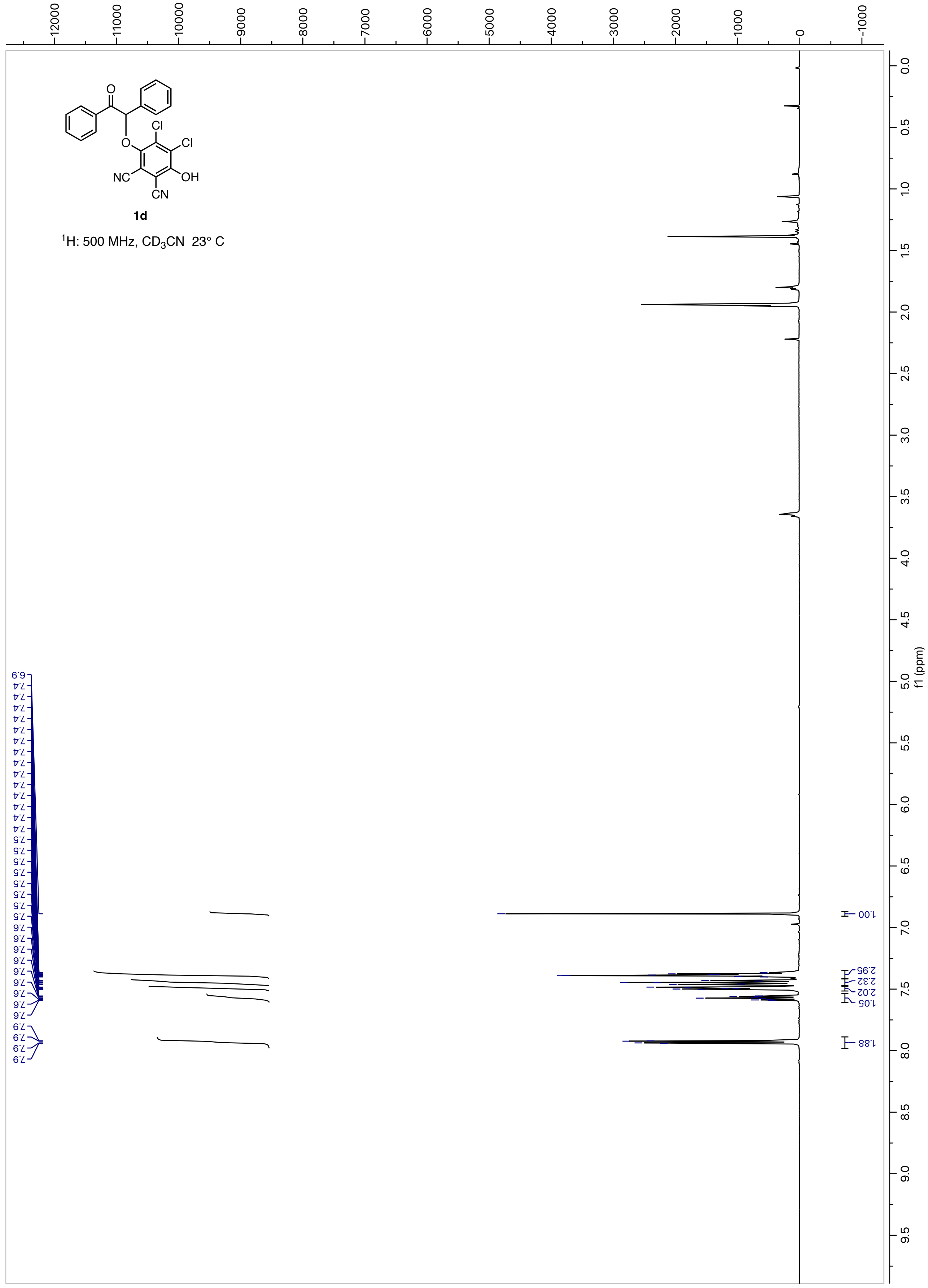

O
Cl
Cl
O
NC
OH
CN
1d
^{1}H: 500 MHz, CD_3CN 23° C
f1 (ppm)
1.00
2.95
2.32
2.02
1.05
1.88

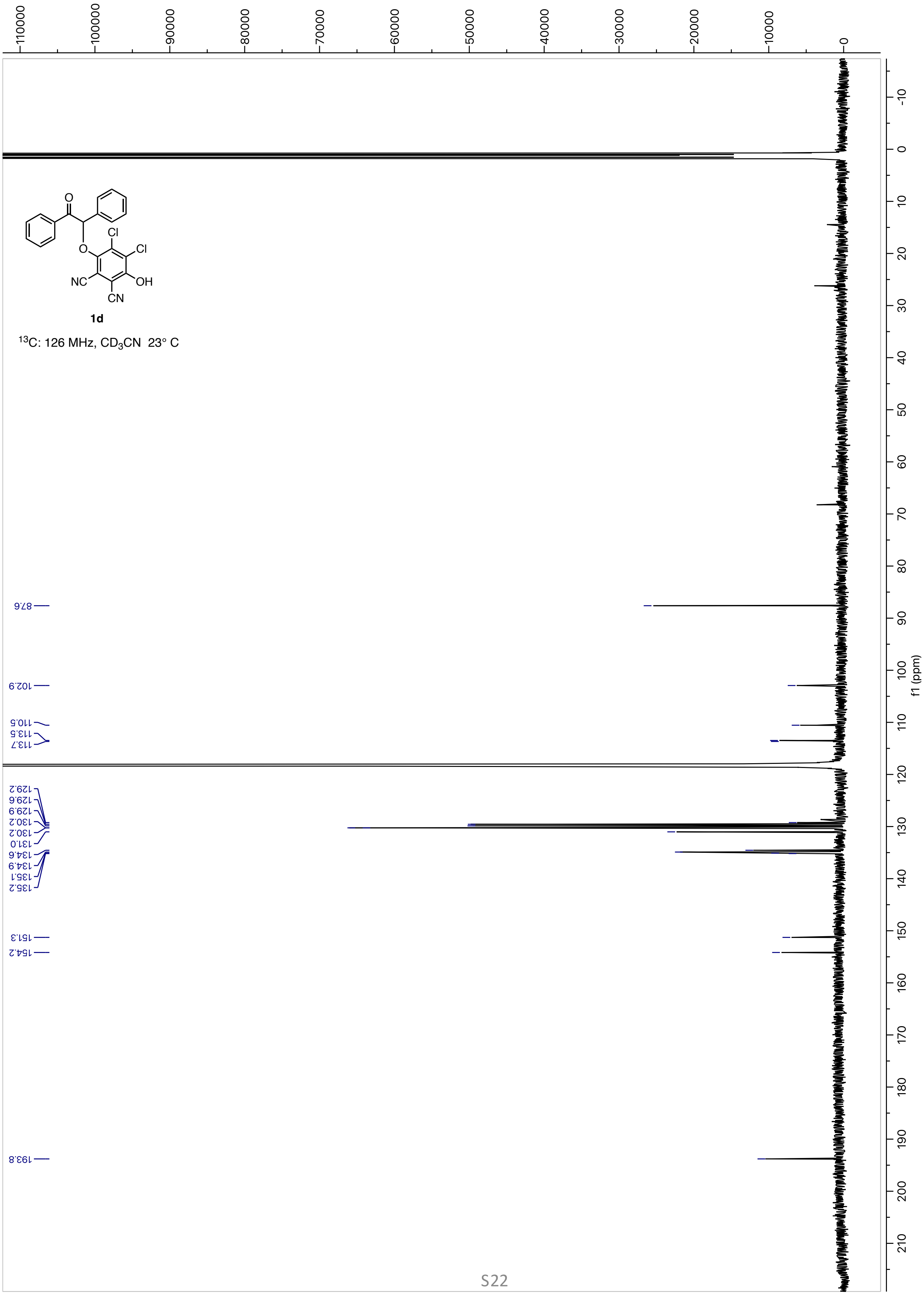
1d
^{13}C: 126 MHz, CD_3CN 23° C
87.6
102.9
110.5
113.5
113.7
129.2
129.6
129.9
130.2
130.2
131.0
134.6
134.9
135.1
135.2
151.3
154.2
193.8
f1 (ppm)
